# Performance improvement of AC servo motor PID control with backlash compensation using a Hybrid Genetic Algorithm–Grey Wolf Optimization approach

**DOI:** 10.1038/s41598-026-64125-3

**Published:** 2026-08-17

**Authors:** Shereen A. Fayad, Mohammed Shaban, Mohamed Attia, Saad A. Mohamed Abdelwahab

**Affiliations:** 1 Department of Electrical, Faculty of Technology and Education, Suez University, P.O.Box: 43221, Suez, Egypt; 2 Egyptian Electricity Transmission Company, Cairo, Egypt; 3 Automation Supervisor at Saint Gobain Glass, Cairo, Egypt

**Keywords:** Experimental investigation, Backlash problem, Programmable logic controller, Human-machine interface and AC servo motor, Electrical and electronic engineering, Renewable energy

## Abstract

This paper presents a comprehensive experimental and simulation-based investigation of intelligent optimization techniques for PID-controlled AC servo drive systems. Three optimization approaches, namely the Genetic Algorithm (GA), Grey Wolf Optimization (GWO), and a Hybrid Genetic Algorithm Grey Wolf Optimization (HGAGWO), are employed to determine the optimal PID controller parameters for high-precision speed and position control under varying operating conditions and load disturbances. The proposed framework combines advanced optimization techniques with experimental validation to provide a reliable assessment of controller performance in practical industrial environments. The experimental platform consists of a programmable logic controller (PLC), a human-machine interface (HMI), an AC servo drive, and a high-resolution external encoder, while MATLAB/Simulink is utilized to develop and validate the dynamic model. Controller performance is systematically evaluated using widely accepted dynamic performance indices, including rising time, settling time, overshoot, steady-state error, and tracking capability under different operating scenarios. The controller optimization study addresses backlash, one of the most significant nonlinearities affecting servo drive accuracy. A backlash compensation strategy is implemented and experimentally verified, demonstrating a noticeable improvement in positioning precision and motion stability. Comparative simulation and experimental results confirm that the optimized PID controllers significantly enhance the transient response, disturbance-rejection capability, tracking accuracy, and overall system robustness compared with conventional PID tuning methods. Among the investigated optimization techniques, the proposed HGAGWO algorithm effectively combines the global exploration capability of GA with the fast convergence characteristics of GWO, producing superior optimization accuracy, faster convergence, and more reliable controller tuning. The close agreement between simulation and experimental results further validates the effectiveness and practical applicability of the proposed methodology. Therefore, the integration of intelligent PID optimization with backlash compensation provides a robust and efficient motion control solution for high-performance industrial servo systems operating under nonlinearities, load variations, and parameter uncertainties.

## Introduction

AC servo motors are often used in industrial settings such as robotics, CNC machines, and packaging systems that need very precise control of speed and position. Control methods have evolved from simple linear methods such as PI and PID to more advanced methods like model predictive control (MPC), adaptive control, sliding mode control, and AI-based schemes. Each of these is meant des to deal with system nonlinearities and disturbances. But we still need industrial-ready technologies that balance dynamic performance under different load conditions, ease of use, and ease of deployment. Recent surveys have given detailed overviews of the newest servo control technologies^1^.

AC servomotors are used in many different fields to control both stationary and moving loads. The main requirement is that they can precisely control position, speed, and torque. Several commercially available control approaches have been developed for AC Servomotors operating under static and dynamic load conditions. However, each of these control strategies has advantages and disadvantages. Although numerous studies have addressed AC servomotor control, relatively little in-depth research has been conducted^1,2^. AC servo motors are preferred in non-linear and dynamic operating environments where an exact feedback response for the reference position is required. The feedback signals are essential for servomotors to respond quickly to on-off actions and maintain their steady state despite unexpected load changes. Various strategies may be used to resolve challenging computational problems driven by process requirements, and industries highly value AC Servomotors due to their precision in responding to dynamic load conditions. The vector control method used with an AC servo motor position Sensor provides less control and functions similarly to a DC motor system. The controller needs information about the magnetic pole location on the motor’s rotor when using vector control with AC servo motors. The controller can estimate both the magnetic pole position and the mechanical position, demonstrating the latter as a first step in the new control technology by employing the AC servo motor perfect position sensor, a less control technique.

The industry placed a high value on precise servo motor control because it reduced error rates in production procedures that demanded extreme precision. To control the servo motor’s rotation angle, PWM was used as the control signal, allowing the motors to operate at maximum torque and high speeds. Servo motors have built-in motor shaft position sensors that are already coupled to the control. In paper^1^, an experimental analysis of the performance of two different types of electric motors, namely permanent magnet synchronous motors (PMSMs) and AC servo motors (ACSMs), is presented for linear positioning applications. The study compares the two motors in terms of their dynamic response, accuracy, and stability under various operating conditions, such as different speeds and load profiles. The results can provide valuable insights into the selection and design of electric motors for linear positioning applications in various industrial and robotic systems. The study presented in^3^ investigated and compared the performance of three control strategies (PI, PD, and PID) for speed control of an AC servo motor through experimental work. The study aimed to evaluate the effectiveness of each controller in terms of steady-state error, rise time, overshoot, and settling time, and to identify the best controller for the given application^3^. The encoder is a device mounted on the AC servo motor shaft that detects the motor’s rotational position and speed, and sends this information to the PLC, which uses it to control the motor’s speed and position accurately. The proximity sensor detects the presence of a pipe and sends a signal to the PLC to initiate the cutting process. With these sensors and the precise control provided by the AC servo motor and PLC, the pipe-cutting machine can cut pipes to very accurate, consistent lengths, making it ideal for industrial applications where precision is critical^1,2^.

Based on this estimated disturbance, the controller adjusts the control action to counteract it and maintain accurate position control. The suggested SMCDE method has been successfully used to control the position of an AC servo motor. The experimental results show that it works well for getting accurate position control even when there are outside disturbances. In terms of accuracy and robustness, the SMCDE outperforms the traditional sliding mode controller, which requires the disturbance bound to be known in advance. In general, the suggested SMCDE method is a new way to get AC servo motors to move exactly where you want them to, even when external forces are at work. This can be very useful for many industrial uses, such as robotics, automation, and manufacturing. Identifying parameters and auto-tuning are two important steps in getting permanent-magnet AC servo motor drives to work well. This process involves identifying and tuning various parameters to achieve optimal motor control. Initially, the electrical parameters of the motor, such as resistance and inductance, which are necessary for tuning the current control loop, are determined. Subsequently, the torque constant and mechanical parameters are determined to tune the velocity and position control loops. The auto-tuning scheme employs various techniques to identify these parameters, eliminating the need for manual intervention and reducing the time and effort required for tuning, while ensuring optimal performance of the motor drive system. The adaptive iterative learning control strategy addresses trajectory tracking for tank gun servo systems with input dead zones and arbitrary initial states. To compensate for the nonzero initial error during the iterative learning controller construction, a time-varying boundary layer is created. The boundary layer is introduced to allow limited deviation from the desired trajectory, avoiding overshooting or instability while adapting to changing conditions. To account for uncertainties and dead-zone nonlinearity, a combination of neural network control and robust control is employed, in which a trained neural network approximates the system’s dynamics and provides feedback to the control system. Robust control employs a control law designed to provide stable performance in the presence of uncertainties and disturbances. The friction compensation scheme for adaptive backstopping control is presented. The friction force is described by the LuGre dynamic friction model, which assumes no uniform variations in friction force, whereas the AC motor control system is designed using a third-order linear dynamic model. Linear controllers are widely used in industrial applications because they perform well in simple situations. In complex situations, intelligent control systems are necessary. PID and fuzzy PID systems were compared, and a mathematical model was developed using MATLAB. They were proven to be important to the outcomes because the system achieved stability more quickly than PI & PID, according to the study technique^3^.

A servo motor is controlled to reach the desired position using various techniques to optimize performance^4^. These methods are categorized as either offline or online, and most identify mechanical and/or electrical parameters during tuning. Establishing all the controllers within a short time is crucial for the scheme to be acceptable in practice. Backlash is always present in the servo system’s transmission, causing a high error rate that degrades the control process. Therefore, a predictive model and a simulation of the automatic control process were developed to ensure system stability and prevent backlash-induced damage. Fuzzy controllers have been used to regulate the backlash motion of servo motors; however, the analysis is complicated by the problem’s nonlinearity. Using genetic algorithms, a set of solutions has been found through a trial-and-error method. Nonlinear electrical loads generate harmonic distortion, which in turn causes a backlash that drives the system out of the dead point. Therefore, a mathematical model and simulation of the electrical load and its distortion, as well as the importance of harmonic processing in reducing backlash, were developed. THD results were recorded before and after the placement of harmonics treatment, demonstrating their significance. However, conventional PID controllers may be limited when dealing with highly nonlinear or uncertain systems. To address these challenges, several studies have proposed enhanced control structures, such as augmented linear and nonlinear PD/PID controllers, which improve tracking accuracy, stability, and robustness^5,6^.

In^7^, the effectiveness of PI controllers in enhancing the control accuracy and stability of DC servo motors in industrial settings was demonstrated. Building on this foundation, the present work extends the investigation to AC servo systems, emphasizing real-time performance evaluation and comparison between PI and PID control approaches.

In^8^, a thorough comparison of servomotor control principles and their real-world applications across industries is presented, highlighting the pros and cons of standard methods such as PI and PID strategies in various situations. The results underscore the importance of selecting control methods based on how the system operates and the application’s needs. This aligns with the ongoing interest in improving servo performance for greater accuracy and stability. These kinds of insights give useful background for more experimental testing in real-time settings.

A thorough and current analysis of Model Predictive Control (MPC) techniques for AC motor drives is given in^9^, focusing on managing operating limits, improving transient performance, and adding AI-based improvements, while also pointing out both industrial readiness and computational problems when looking at uses in precision mechatronic systems, PMSM motors, and multi-objective current control. In^10^, a composite MPC architecture for PMSM servo systems is presented^10^, which includes an Extended State Observer (ESO) to account for current loop execution delays and demonstrates improvements such as. Rapid transient response, resilience against load disruptions, and decreased tracking error. An event-triggered finite-control-set MPC (FCS-MPC) architecture for PMSM drives is proposed in^11^, along with a sliding-mode observer (SMO) for estimating disturbances. The event-triggering technique maintains good control precision under dynamic disturbances while lowering the computational and communication burden.

To improve flexibility and resilience in nonlinear control tasks, several high-impact modern PID variants have been introduced: Sigmoid-based PID (SPID) uses a sigmoid function to dynamically adjust gains, thereby speeding up settling in voltage-regulator systems^12^. Sigmoid-based Fractional-Order PID (SFOPID) uses a dandelion optimizer to improve dynamic response by combining sigmoid gain scheduling with fractional calculus^13^. Data-driven BELBIC-PID incorporates the principles of brain emotional learning into a PID framework for robust control of MIMO systems^14^, and hardware Implementations of FOPID Controllers demonstrate practical fractional-order PID applications using FPGA and NI myRIO platforms^15^.

Although newer PID types are more flexible and better handle nonlinear problems, traditional PID controllers remain widely used in industry because they are reliable, easy to integrate with PLCs such as the Siemens S7-1200, require little computing power, and are standardized. In this study, their performance is further improved by GA, GWO, and HGAGWO for intelligent tuning of PID controllers. This combines industrial-strength with ease of use and intelligent tuning to deliver better dynamic performance while ensuring it is fully feasible for real-time PLC-based applications.

They investigate the performance accuracy of various control methods for AC servo motors, including PID controllers, using both theoretical and experimental approaches. The high-speed servomotor typically drives the transmission, completing the energy conversion by driving the load. This was the construction of the conventional PMSM servo system. Gears and ball screws are commonly employed in transmission mechanisms and inherently exhibit backlash nonlinearity^16,17^.

Recent advances in motor-drive control have increasingly focused on improving robustness and tracking accuracy under parameter uncertainties and external disturbances. Their results highlighted the importance of advanced control frameworks in maintaining stability and tracking precision in practical motor-drive applications. Nevertheless, such approaches often rely on sophisticated control structures and accurate system modeling, which may increase implementation complexity in industrial servo systems^18^. Senseless control techniques have attracted significant attention in PMSM drive systems due to their ability to reduce hardware requirements while maintaining reliable operation. Advanced observer-based approaches have achieved accurate rotor position estimation and improved low-speed performance, thereby enhancing efficiency and reliability in modern motor-control applications^19^. Machine-learning-based approaches have recently been introduced to improve prediction and control performance in PMSM drives. Differential neural network techniques have shown promising results in current prediction and adaptive learning under dynamic operating conditions, offering new opportunities for intelligent motor-drive control and performance enhancement^20^.

Recent studies have demonstrated the effectiveness of reinforcement learning-based model predictive control and adaptive fuzzy PID control in enhancing the dynamic performance, tracking accuracy, and robustness of motor-drive systems. These intelligent control strategies highlight the growing trend toward integrating advanced optimization and adaptive techniques to improve the performance of modern electromechanical systems^21,22^.

Table 1 summarizes the most important prior research on controlling servo motors, including the system components, the control methods used, and the areas where they can be applied. It shows how PI/PID controllers have evolved into MPC methods and intelligent PID variants, offering better performance and a wider range of applications. The proposed study utilizes a GA, GWO, and HGAGWO PID controller to integrate industrial reliability with improved real-time performance in PLC-based control systems.

**Table 1:** Comparative summary of previous studies on servo motor control strategies.

Ref.	Controlled system	Controller / optimization	Implementation	Main contribution
^7^	AC Servo Motor	PI	PLC	Industrial speed regulation
^8^	AC Servo Motor	PID	Experimental	Speed and position tracking
^9^	PMSM	MPC	Simulation	Constrained predictive control
^10^	PMSM	Composite MPC + ESO	Simulation	Disturbance rejection
^11^	PMSM	Event-triggered FCS-MPC	Simulation	Reduced computational burden
^12^	Servo System	Sigmoid PID	Simulation	Adaptive gain scheduling
^13^	AVR	Sigmoid FOPID	Simulation	Improved dynamic response
^14^	MIMO Servo	BELBIC-PID	Simulation	Intelligent robust control
^18^	Hub Motor	Hybrid Robust Control	Experimental	Robust tracking under uncertainties
^19^	PMSM	Senseless Control	Experimental	Zero-speed position estimation
^20^	PMSM	Differential Neural Network	Simulation	Intelligent current prediction
^21^	PMSM	RL-MPC	Simulation	Learning-based predictive control
^22^	Pneumatic Manipulator	Adaptive Fuzzy PID	Experimental	Adaptive PID tuning
This work	AC Servo Motor + Siemens S7-1200 PLC	HGAGWO-Optimized PID + Backlash Compensation	Simulation & Experiment	Real-time PLC implementation with intelligent PID tuning

In automation, robotics, automotive, and industrial applications, backlash is a crucial component that influences the servo system’s dynamic tracking and steady-state performance. Backlash research is challenging complicate servo system control problems. Consequently, in-depth research on backlash nonlinearity for servo systems is required. Nonlinearities such as nonlinear friction, backlash, and external disturbances impede control performance. The transmission device’s backlash, which degrades transmission performance, was one such nonlinearity. Some cutting-edge control techniques have been developed to lessen the effects of blowback, such as robust control, adaptive control, and sliding mode control. Additionally, artificial intelligence technologies, such as neural networks and fuzzy logic control, have been utilized to compensate for nonlinearities due to their ability to approximate and learn nonlinear functions.

The following is a summary of this study’s primary contributions:

- A comprehensive review of conventional and advanced control techniques for AC servo motors is presented, highlighting existing research gaps, particularly in practical PLC-based implementations and backlash-affected systems.
- A HGAGWO approach is developed for optimal tuning of PID controller parameters, and its performance is benchmarked against standalone GA and GWO techniques.
- The proposed optimized PID controllers are implemented on a Siemens S7-1200 PLC, ensuring industrial feasibility and real-time applicability of the control strategy.
- Extensive experimental validation under real-time operating conditions is conducted, demonstrating significant improvements in dynamic response, reduced steady-state error, reduced overshoot, and quicker settling time when compared to traditional tuning techniques.
- Backlash compensation techniques are integrated with the optimized control framework, leading to enhanced positioning accuracy and improved robustness in the presence of nonlinearities.
- A combined simulation and hardware-based validation framework is established, effectively bridging the gap between theoretical design and practical industrial implementation.

The primary aim of this study is to conduct an in-depth analysis of AC servo motor control performance using PID control strategies under realistic operating conditions. In a real-time setting, the system uses a programmable logic controller (PLC), a human-machine interface (HMI), and a servo drive. An external encoder provides accurate feedback. The study focuses on regulating both the motor’s position and velocity, and assesses system performance under diverse dynamic and static load conditions. To assess how well a control system performs, we examine key performance indicators such as rise time, settling time, overshoot, and steady-state error. Through validation, the experimental outcomes are compared with simulation data generated in MATLAB. The study also suggests examining real-world problems, such as the backlash effect, and proposes a way to pay people to improve control accuracy and stability. The goal of this work is to connect theoretical modeling with real-world use by giving a practical evaluation of control methods for AC servo motor systems.

## Mathematical modelling and experimental setup of the AC servo control system

This study develops and validates an advanced position and speed-control system for an AC servo motor through simulation and real-time experimental implementation. The system is modeled as a second-order dynamic system incorporating dual feedback loops for position and speed control. The mathematical model of the AC servo motor is based on its electrical and mechanical properties, such as inertia, viscous damping, back electromotive force, and torque constants. Based on this model, a PID controller is designed and optimally tuned using an HGAGWO approach, along with standalone GA and GWO techniques, to ensure robust, high-performance control under varying operating conditions. The experimental setup includes an AC servo motor with a servo drive, a human–machine interface (HMI), and a programmable logic controller (PLC). This allows the proposed control strategies to be implemented right away. The optimized PID controllers are employed to regulate both position and speed, and their performance is comparatively evaluated in terms of dynamic response and steady-state accuracy. We use MATLAB to simulate the system and verify the experimental results. Furthermore, the study addresses the nonlinear effects of backlash in servo systems by incorporating an effective compensation strategy into the control framework. This integration has greatly improved. AC servo motors are widely utilized in high-precision industrial applications such as CNC machines, robotics, and automated manufacturing systems due to their high efficiency and superior dynamic characteristics. Accurate feedback signals, such as position, speed, and torque, are crucial for ensuring the system responds quickly and remains stable when the load changes suddenly. So, the suggested hybrid optimization-based PID control scheme, with backlash compensation and real-time implementation, is a reliable approach for controlling advanced industrial motion.

**Fig. 1:**
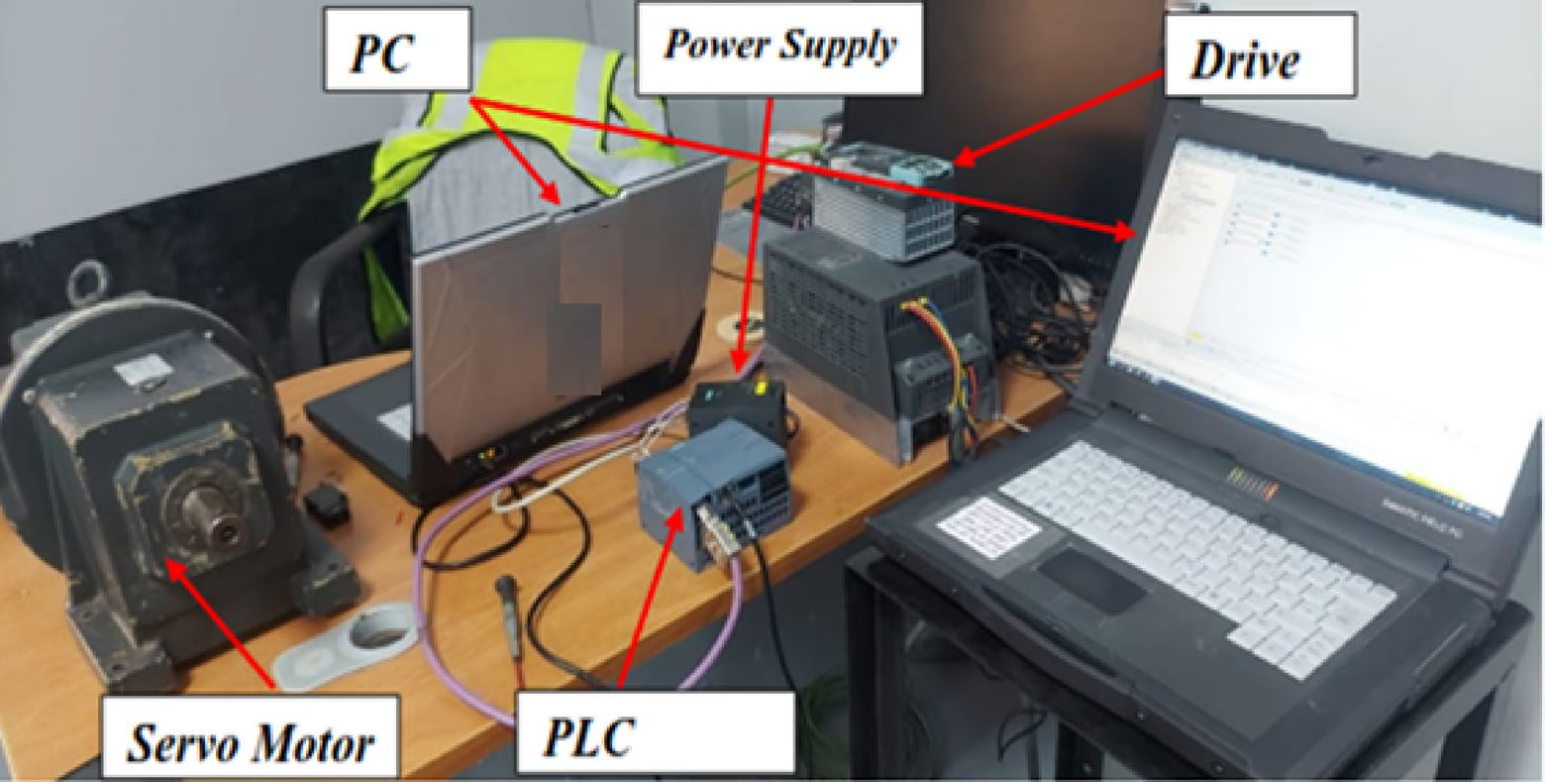
Experimental work of the proposed system.

Figure 1, actual experimental work using PLC Siemens S7-1200, Siemens HMI, Siemens Servo Drive, external encoder, Siemens PG, and Siemens servo motor. The Siemens PLC S7 1200, servo motor, used power supply, and used external encoder are all depicted. All electrical components are connected in order, as shown in the figure. In step two, the driving parameters are set up. In step three, the HMI programmer is set up. In step four, the PLC programmer is made, and the PID controller is built. After completing all prior steps, turn on the system, set the entry speed and position on the HMI, and automatically tune the PID controller. Then you can also manually change the PID parameter and see what happens. This experiment was different because it used two PID controllers: one built into a PLC and the other built into the Drive itself. By using an external encoder directly connected to the load to measure the actual load position, this method improves accuracy and transitions the system from a semi-closed loop to a fully closed loop.

Based on the information provided, the figure appears to show an experimental setup using various Siemens components, including a PLC (S7-1200), an HMI, a Samanic Servo Drive, an external encoder, a PG, and a servo motor. The setup involves connecting all the electrical components as shown in the figure and preparing driving parameters in step two. In step three, you get an HMI programmer ready. In step four, you make a PLC programmer, and in step five, you make a PID controller. After finishing all the steps, the system is turned on, and the HMI is set to the entry speed and position. The PID controller is then automatically tuned, or the parameters can be examined and adjusted manually. One interesting thing about this experiment is that it uses two PID controllers, one built into the PLC and the other built into the Drive itself. This method enhances accuracy and converts the system from a semi-closed loop to a fully closed loop by utilizing an external encoder connected directly to the load to measure the actual load position.

**Fig. 2:**
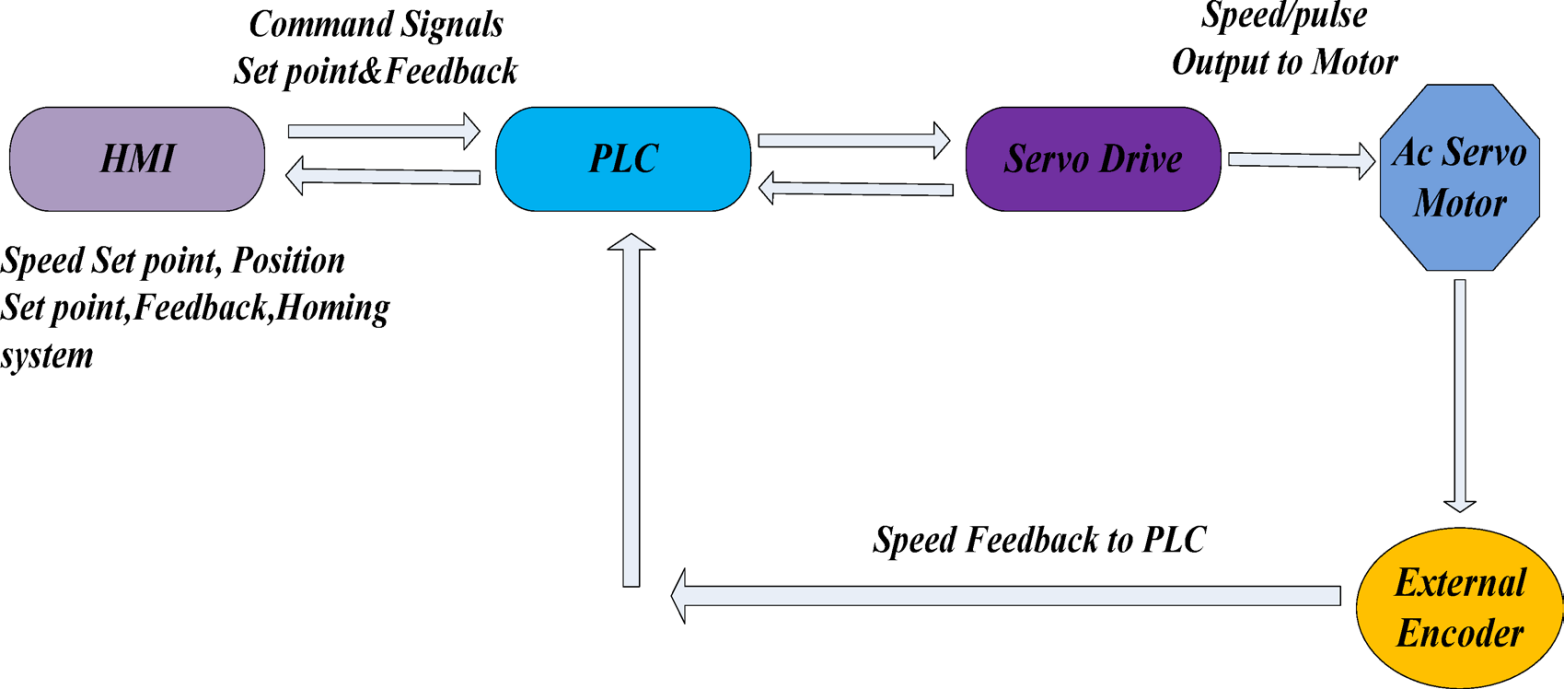
Block diagram of the control system.

The block diagram of the proposed system is shown below. The input devices used to provide input signals to the PLC are the selector and the external encoder, as shown in Fig. 2. The selector is used to turn the system on or off. The external encoder is used to measure output speed and position. The PLC output signal is used to control servo drives, and the HMI is used for Entry data such as speed set point, position set point, and present position feedback. However, based on the description provided, that provide input signals to the PLC. The selector is used to turn the system on and off, while the external encoder measures the output speed and position of the system. The PLC output signal is then used to control the servo drives. Additionally, an HMI is used to enter data, such as speed and position setpoints, and to provide feedback on the current position. The Siemens AC servo motor (3.77 kW, 12 Nm, 3000 rpm), Siemens S7-1200 PLC, 7″ HMI for operator interaction, and an external 2048 P/R encoder for accurate speed and position measurement are among the components of the experimental setup that are listed in Table 2. These components form a useful framework used to verify the proposed control approach in real-time scenarios. Table 3 lists the mechanical and electrical characteristics of the used AC servo motor, including the torque constant (1.59 Nm/A), damping coefficient (0.0065), inertia (0.0055 kg·m²), armature voltage (331 $\:{\mathrm{V}}_{\mathrm{r}\mathrm{m}\mathrm{s}}$), resistance (0.34 Ω), and inductance (7 mH). These characteristics are the foundation for simulation, controller tuning, and experimental validation, and were essential in creating the mathematical model of the servo system.

**Table 2:** Parameters of experimental work.

Parameters	Values
Siemens AC Servo Motor 1FK7100-5AF71-1DU5-Z	Power = 3.77 KW Current = 8 A. Speed = 3000 rpm Torque = 12 NM
Siemens PLC	PLC 1200
Siemens HMI	Touch 7″
Sick external encoder	2048 P/R
Selector siemens	Normally open

**Table 3:** AC servo motor parameters.

Parameters	Values
Armature voltage $\:{V}_{a}$	331 $\:{\mathrm{V}}_{\mathrm{r}\mathrm{m}\mathrm{s}}$
Armature resistance $\:{R}_{a}$	0.34 Ohm
Armature inductance$\:\:{L}_{a}$	7Mh
Motor inertia $\:\:{J}_{m}$	0.0055 kg-m2
Motor viscous damping Coefficient $\:{B}_{m}$	0.0065
Motor torque constant coefficient$\:\:{K}_{t}$	1.59 Nm/A
Rated motor power	3.77 kW
Rated motor current	8 Arms
Maximum motor current	37 Arms
Rated motor torque	12 Nm
Rated motor speed	3000 rpm
Maximum motor speed	5000 rpm

## Advanced controller of AC servo motor

**Fig. 3:**
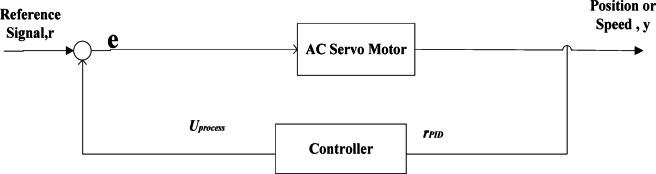
Controller of the AC servo motor.

A comparative evaluation of PID control strategies in terms of rise time, settling time, and overshoot is presented in Fig. 3 for both speed and position control of the AC servo motor under various operating conditions. The comparison includes conventionally tuned PID as well as optimized PID controllers based on GA, GWO, and the proposed HGAGWO approach. The results clearly show that the optimized PID controllers work much better than the regular PID controllers, especially when the load changes or the operating conditions change. This is because they are better at handling nonlinear system dynamics. PID control is still one of the most popular control methods because it is simple and effective. The proportional term gives an immediate response to the error, the integral term gets rid of steady-state error, and the derivative term makes the system more stable by predicting how fast the error is changing. In this study, the PID controller parameters are optimally tuned using GA, GWO, and the proposed HGAGWO approach. This lets the control gains be adjusted automatically and intelligently to get better dynamic performance, higher accuracy, and more robustness. Backlash compensation is also built into the control framework to lessen the negative effects of mechanical nonlinearities. This makes the system more accurate and responsive, especially when the load changes. Figure 3 shows the closed-loop control structure of the proposed system. The error signal, e(t), is generated by comparing the reference input, r(t), with the measured output, y(t), of the AC servo motor. This error is processed by the optimized PID controller, whose parameters are tuned using the GA, GWO, or the proposed HGAGWO algorithm, to generate the control signal applied to the servo drive. Through continuous feedback, the controller minimizes the tracking error and improves the dynamic response of the system. The PID controller consists of proportional (Kp), integral (Ki), and derivative (Kd) actions, which respectively improve the response speed, eliminate steady-state error, and enhance the transient response by reacting to the rate of change of the error signal. Owing to its simple structure, ease of implementation, and reliable performance, the PID controller remains one of the most widely used control strategies in industrial servo systems. The controller output is obtained by combining the proportional, integral, and derivative terms, as described by Eqs. (1)–(4). Figure 1 illustrates the overall control architecture adopted in this study. The proposed system employs a cascaded dual-loop configuration consisting of an outer position control loop and an inner speed control loop. The outer-loop PID controller is implemented in Siemens S7-1200 PLC and generates speed reference according to the position error. This reference is transmitted to the servo drive, where the embedded PID controller regulates the motor speed. Position feedback is provided by an external encoder mounted on the load shaft, while the servo drive utilizes its internal encoder for speed regulation. This dual-feedback configuration improves position tracking accuracy and disturbance rejection while maintaining effective coordination between the PLC-based position controller and the drive-based speed controller^18,19^. However, if PI controller is used the control signal will be the same in moment $\:{t}_{1}:u\left({t}_{1}\right)$ Proportional will be proportional to error in $\:{t}_{1}\::$

1 $$\:{u}_{p}\left({t}_{1}\right)=\:{K}_{e}\left({t}_{1}\right)$$

Integral part of the signal will be proportional to the area under error curve till moment^23,24^ $\:{t}_{1}\::$.

2 $$\:{u}_{1}\left({t}_{1}\right)=\frac{K}{{T}_{i}}\:{\int\:}_{0}^{{t}_{1}}e\left(t\right)dt\:\:\:\:\:$$

**Fig. 4:**
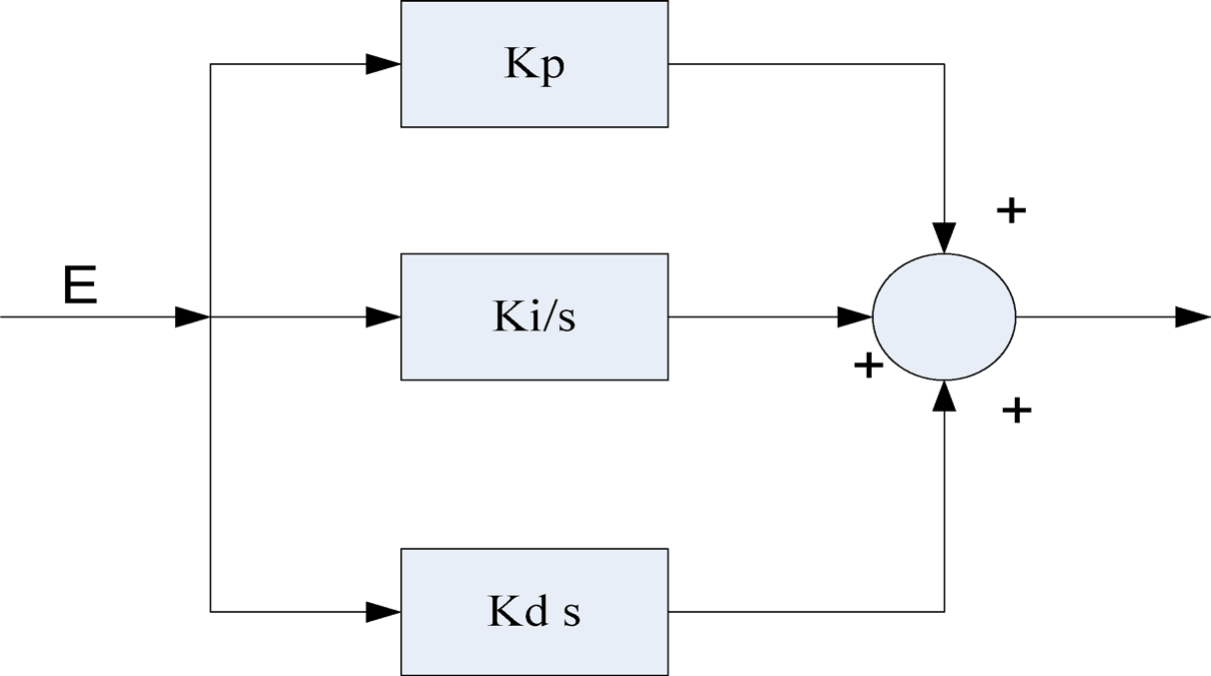
Parallel PID controller.

The control signal of PID controller is:

3 $$\:u\left(t\right)=K\left[e\left(t\right)+\:\frac{1}{{T}_{i}}{\int\:}_{0}^{t}e\left(t\right)dt+\:{T}_{d}\:\frac{{d}_{e}\left(t\right)}{dt}\right]$$

or

4 $$\:u\:\left(t\right)={K}_{e}\left(t\right)+{K}_{i}{\int\:}_{0}^{t}e\left(t\right)dt+{K}_{d}\frac{{d}_{e}\left(t\right)}{dt}$$

where:

$\:{K}_{i}=\frac{K}{{T}_{i}}$ gain (reset) of integral part of the controller,

$\:{K}_{i}=\frac{K}{{T}_{i}}$ gain (reset) of integral part of the controller,

The PID controller’s derivative component is proportional to the error signal’s prognosis at time $\:t\:+\:{T}_{d}$ where$\:{T}_{d}$ is the controller’s derivative time constant. Each action parameter (kp, ti, td) in the parallel equation is independent of the others. At first appear, this could seem advantageous since, as Fig. 4 illustrates, each disk of the control unit only needs to impact on one aspect of its operation. The PID controller having all the required dynamics, it may be concluded: quick response to a shift in the controller input (D mode), an increase in the control signal to bring the error closer to zero (I mode), and appropriate action within the control error area $\:e\left(t\right){\:<e}_{0}$to get rid of oscillations (P mode)^23^,^24^].

## Proposed algorithms

### The genetic algorithm (GA)

Finding the ideal PID controller parameter values to enhance the system’s transient response is the main goal of the GA. The algorithm considers the maximizing of an objective function to do this. In order for the best person to create an optimal controller, this objective function offers a way to assess the PID controller’s performance using the established gain settings^23^. The genes in the PID controller design problem are the controller $\:{K}_{P},{K}_{i},and\:{K}_{d}$ gain values that need to be ascertained. Every chromosome has all the genes required to define a trial solution in a unique way. The error criterion is used to assess each chromosome’s fitness and serves as the basis for chromosome selection in the next generation^24^.

During the optimization phase, the genetic algorithm makes use of both the necessary poles and the real closed-loop poles. Closed-loop poles are identified in each generation based on the controller parameters. The tuning process utilizes these closed-loop poles to identify the optimal PID controller values. We stop the operation after 100 generations and utilize a population size of 20. A single-point crossover procedure was used in the algorithm. The average of the satisfactory answers is used to determine the neighbors of advantageous chromosomes. In every generation, a fitness function, considered the opposite of the error function, evaluates all solutions in the population^24^. By comparing the actual and necessary closed-loop pole locations, Error E can be computed. It can be expressed as

5 $$\:E=\:\propto\:\left({E}_{os}\right)+\:\beta\:\left({E}_{Ts}\right)+\:\gamma\:\left({E}_{Td}\right)+\:\delta\:\left({E}_{on}\right)+\:\lambda\:\left({E}_{of}\right)$$

Where:

$\:{\mathrm{E}}_{\mathrm{o}\mathrm{s}}$ : represents the percentage of overshoot error.

$\:{\mathrm{E}}_{\mathrm{T}\mathrm{s}}$ is the settling time error.

$\:{E}_{Td}$ : is the error in peak time.

$\:{\mathrm{E}}_{\mathrm{o}\mathrm{n}}$ and $\:{E}_{of}\:$: are the errors in the location of the real poles.

The weight age factors are represented by the variables α, β, γ, δ, and λ. The best PID controller parameters to attain the necessary closed-loop response can be found by modifying these variables. Highly fit solutions are chosen following the fitness assessment, and the crossover operation is carried out to remove springs. Any random value can be changed to cause a mutation. The solution is slightly altered as a result. Replace the entire population with the offspring, the best solution, and the neighbor of good solutions, following these ways of reproduction. To find a better answer, carry on with the evaluation, reproduction, etc., procedure for a predetermined number of generations. When predefined termination conditions are not met, algorithms can be summed up as initializing the population.

1. Use the fitness function, which can be the inverse of the error function, to evaluate each of these solutions.
2. Choose a few very suitable options.
3. To create offspring, pair them as parents and carry out crossover operations.
4. Modify a random answer significantly to carry out the mutation.
5. Using the average of good solutions, come up with some solutions.
6. Keep the best option.
7. Replace the entire population with the best answer, these offspring, and the neighbors of good solutions.

**Fig. 5:**
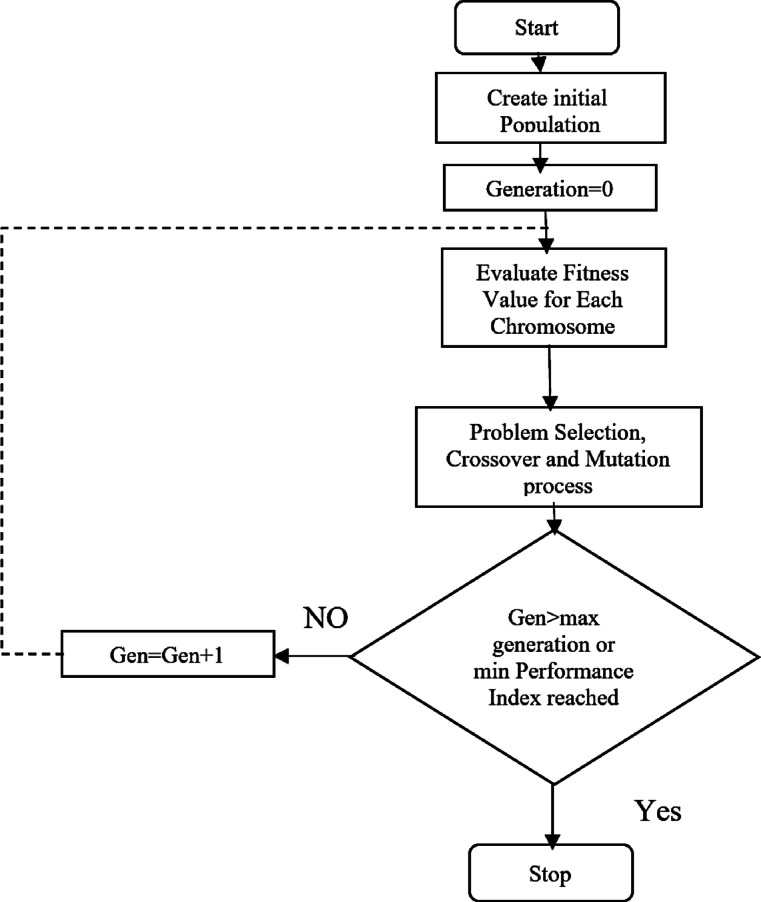
Flow chart of genetic algorithms.

Figure 5 shows the GA architecture, which gives a visual representation of how the algorithm works and how it flows. This framework shows that GA is both iterative and evolutionary, highlighting its ability to explore different solution spaces and converge toward optimal solutions through successive generations^25–28^

### The algorithm for grey wolf optimization

The Gray Wolf Optimization (GWO) algorithm is a sophisticated approach inspired by gray wolves’ predatory behavior. in earlier brilliant algorithms, the ideal solution is represented by the prey, whereas each gray wolf’s position represents a possible solution. To find the optimal solution, gray wolves are ranked according to the degree of fitness function value. α wolves, β wolves, and δ wolves are the gray wolves with the highest fitness function value. The remaining wolves are ω wolves, whereas α, δ, and β wolves are nearer the prey’s possible location. The hierarchy of gray wolves is crucial to the predation process. α wolves lead the group in encircling their victim, followed by β and δ wolves who assault it, and ω wolves who help finish the attack before capturing it^29^. This enables precise manipulation of PID settings^29,30^.

The following components make up the gray wolf optimization algorithm’s mathematical model^29^:

Surround prey:

6 $$\:D=\left|C.{X}_{P}\left(t\right)-X\left(t\right)\right|$$

7 $$\:X\left(t+1\right)={X}_{P}\left(t\right)-A.D\:\:$$

Equation (7) is the gray wolf’s location update formula, while Eq. (6) shows the distance between the individual gray wolf and its prey. $\:{X}_{P}$and *X* are the position vectors of the prey and the gray wolf, respectively; t is the current iteration; and A and C are coefficient vectors. The following are A and C calculating formulas^29,30^:

8 $$\:A=2a.\:{r}_{1}-a\:$$

9 $$\:C=2\:{r}_{2}\:$$

The convergence factor among them is α. The modulus of$\:\:{r}_{1}and\:{r}_{2}$ takes a random number between 0 and 1 as the number of iterations drops linearly from 2 to 0.

Track hunting:

10 $$\:\left\{\begin{array}{c}{D}_{\alpha\:}=\:\left|{C}_{1}.\:{X}_{\alpha\:}-X\right|\\\:{D}_{\beta\:}=\:\left|{C}_{2}.\:{X}_{\beta\:}-X\right|\\\:{D}_{\delta\:}=\:\left|{C}_{3}.\:{X}_{\delta\:}-X\right|\end{array}\right\}$$

Among them, $\:{\mathrm{D}}_{{\upalpha\:}}$, $\:{\mathrm{D}}_{{\upbeta\:}}$, and $\:{\mathrm{D}}_{{\updelta\:}}$ represent the distance between α, β, and δ and other individuals, respectively; $\:{\mathrm{C}}_{1},{\mathrm{C}}_{2}\:\mathrm{a}\mathrm{n}\mathrm{d}{\mathrm{C}}_{3}$ are random vectors; X is the current gray wolf position.

11 $$\:\left\{\begin{array}{c}{X}_{1}=\:{X}_{\alpha\:}-{A}_{1}.\left({D}_{\alpha\:}\right)\\\:{X}_{2}=\:{X}_{\beta\:}-{A}_{2}.\left({D}_{\beta\:}\right)\\\:{X}_{3}=\:{X}_{\delta\:}-{A}_{3}.\left({D}_{\delta\:}\right)\end{array}\right\}$$

12 $$\:X\left(t+1\right)=\frac{{X}_{1}+\:{X}_{2}+{X}_{3}}{3}\:$$

**Fig. 6:**
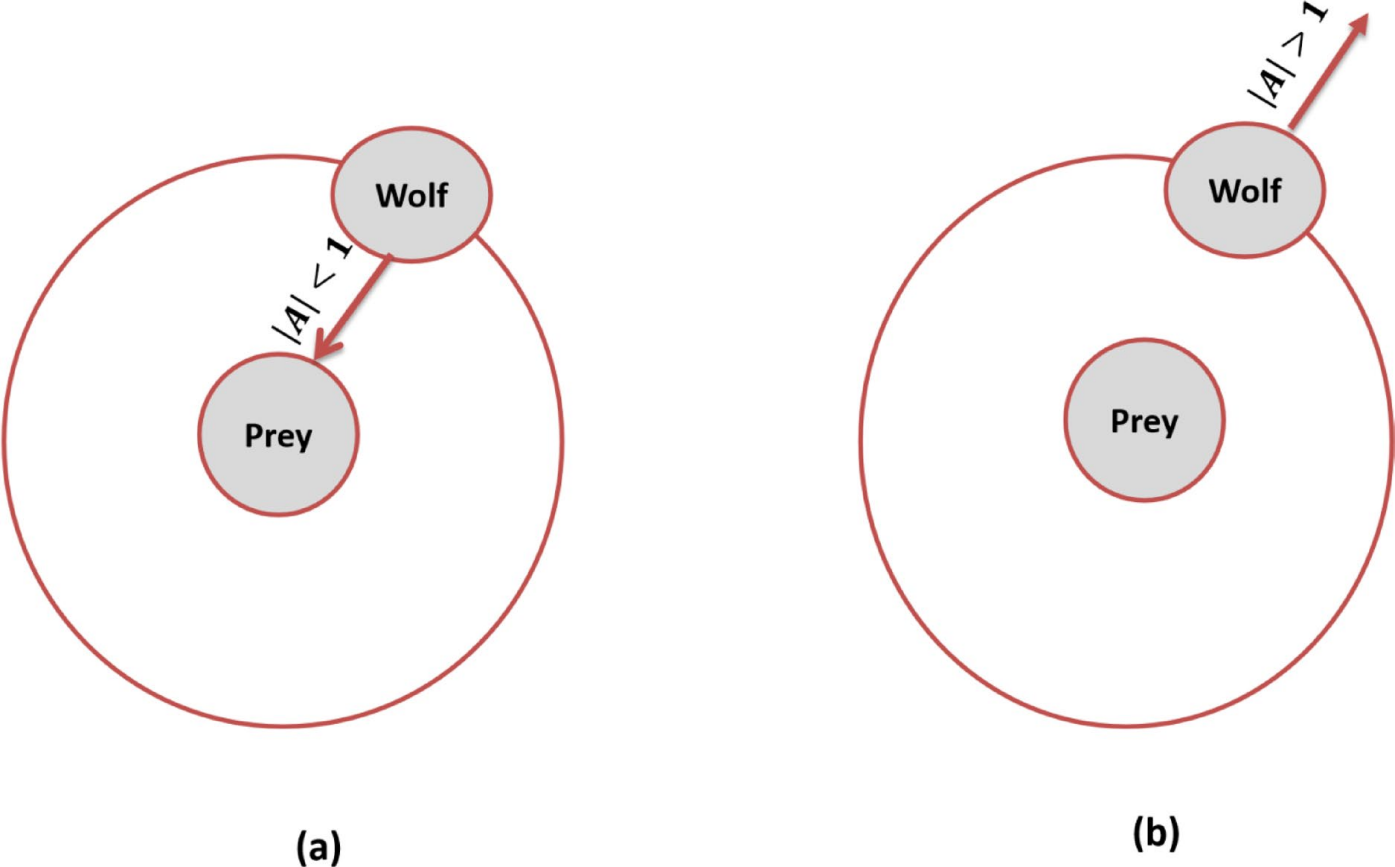
shows how wolves seek and attack their prey: (**a**) wolves attack their victim; (**b**) gray wolves split from their prey.

Equation (12) specifies the end position of ω, while Eq. (11) specifies the wolf pack members’ step length and direction in relation to α, β, and δ, respectively. Go after the prey:

The gray wolf completes the hunt by attacking when the prey stops moving. The value of an is gradually decreased to mimic approaching prey; hence, the fluctuation range of A is likewise decreased. Put otherwise, throughout the iterative process, when the value of a declines linearly from 2 to 0, the equivalent value of A likewise changes in the interval (-a, a). The gray wolf’s next position can be anywhere between its current position and the position of its prey when the value of A falls within the interval, as Fig. 6 illustrates. When $\:\left|A\right|<1$The wolves attack their prey. When $\:\left|A\right|>$,The gray wolf is separated from its prey, hoping to find a more suitable prey^30^.

**Fig. 7:**
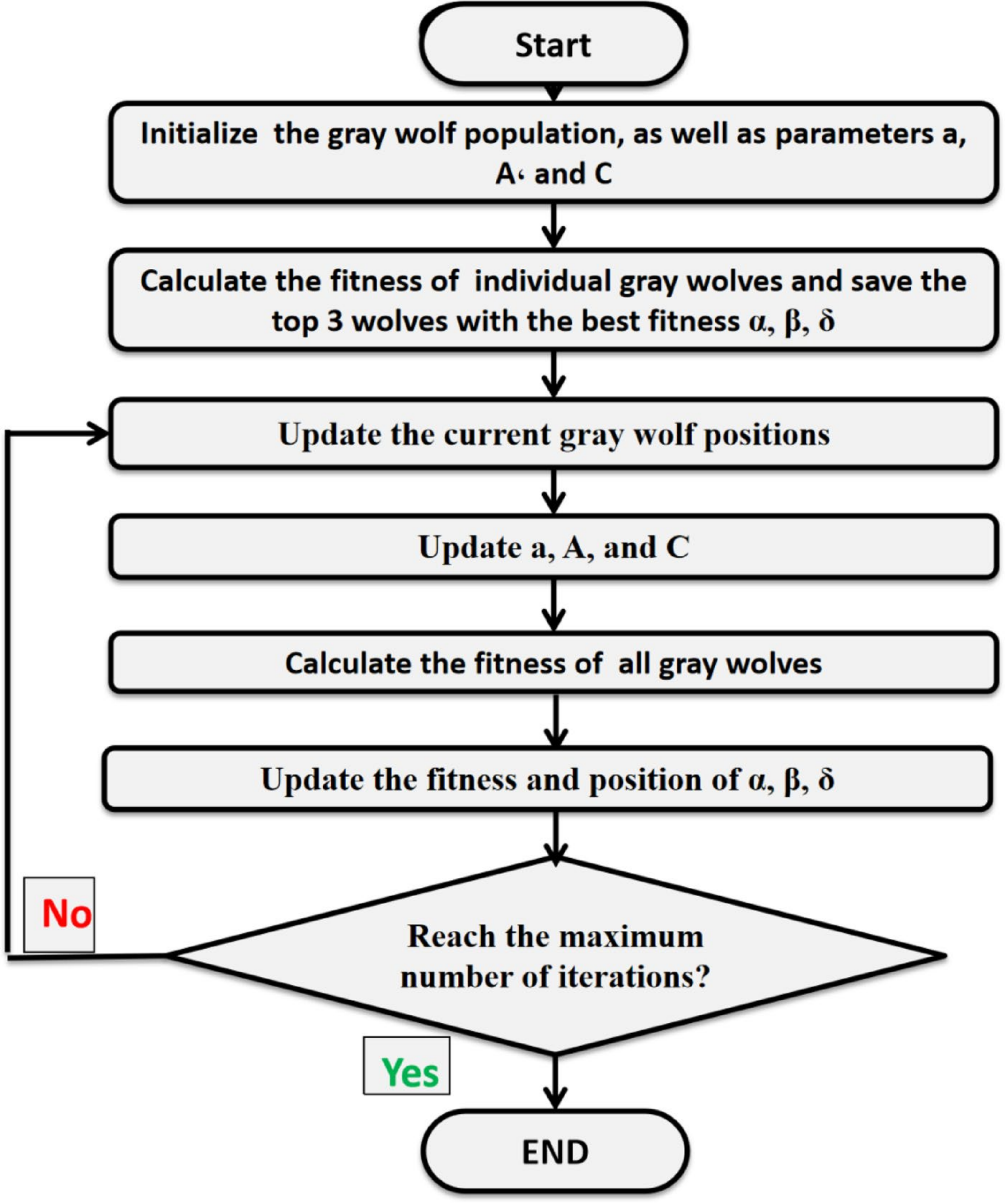
The GWO process.

Figure 7 illustrates the procedural flowchart of the Grey Wolf Optimizer (GWO) algorithm. The optimization process commences with the random initialization of the grey wolf population and the primary control parameters (a, A, and C). The hierarchy of leadership is established by identifying the three best candidates $\:\left(\propto\:,\beta\:\:and\:\delta\:\right)$, which guide the search process. Throughout the iterative phase, the positions of the remaining search agents are dynamically updated based on the leaders’ coordinates. This cycle repeats, refining the fitness values and updating the parameters until the maximum number of iterations is reached. This structured approach ensures a robust transition from exploration to exploitation, ultimately converging toward the global optimum.

### Hybrid GWO and GA

**Fig. 8:**
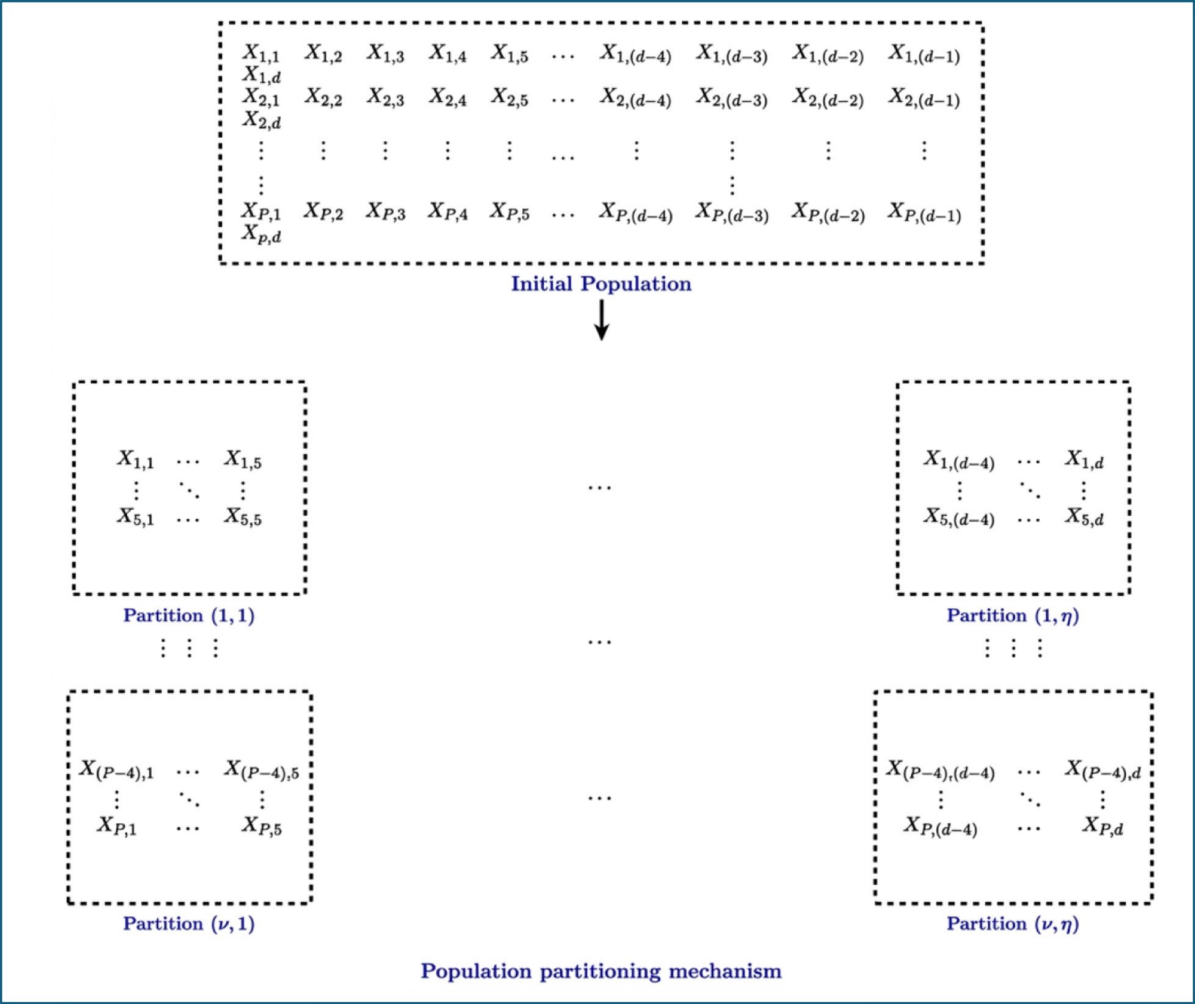
HGAGWO of population partitioning procedure.

The suggested approach performs better when the two techniques are combined, as shown by the numerical results and Fig. 3. Because the HGAGWO algorithm is based on three phases, it is reliable and able to precisely determine PID controller parameters, which are frequently challenging to address. The suggested technique uses the grey wolf optimizer algorithm, which does a decent job at striking a balance between exploration and exploitation, after randomly creating the starting population. The suggested technique uses the grey wolf optimizer algorithm to investigate the search space and take advantage of the promising region with a good solution. However, the grey wolf optimizer algorithm suffers from early convergence and gets stuck in a local minimum in large-scale optimization problems, such as those with dimensions more than 100. As seen in the following subsection, we divide the population into subpopulations and use the dimension reduction process to reduce the problem size to address large dimensionality problems^31–33^. To address some of the difficulties arising from the increasing size and complexity of PID controller tuning parameters for servo motor control, this study presents a unique hybrid algorithm combining the Gray Wolf Optimizer and a genetic algorithm, called HGAGWO. This algorithm aims to ensure comprehensive search space coverage and good convergence towards a global optimum (while avoiding local optimums) even as the problem dimensions increase. HGAGWO achieves this through ensemble partitioning. Each partition is considered a subspace in the search process and contains a defined number of individuals. Search diversity is maintained by using different subspaces. The population partitioning procedure is shown on Fig. 8. The population is divided into multiple groups in Fig. 4 (Pi, j, i = 1…, v; j = 1…, η). To increase search diversity and prevent the premature convergence of the standard grey wolf optimizer algorithm, we apply GA operators (crossover, mutation) on each subpopulation.

Although the PID tuning problem involves only three controller PID parameter, the optimization objective is characterized by a nonlinear and multimodal search landscape due to the coupled dynamics of the AC servo motor, backlash effects, and multiple transient performance indices. Consequently, conventional optimization algorithms may converge prematurely to local optima depending on the initial population and search trajectory. The proposed HGAGWO algorithm combines the exploration capability of the Genetic Algorithm with the exploitation mechanism of the Grey Wolf Optimizer to achieve a better balance during the search process, leading to more reliable PID parameter optimization and improved control performance.

To improve population variety, the suggested HGAGWO algorithm incorporates GA operators straight into the GWO gravitational search procedure. In contrast to sequential hybrid models, each iteration applies the GA mechanisms (crossover and mutation) after the lead wolves) α, β, and δ) are used to update the wolves’ placements. This guarantees that the stochastic exploration of GA and the social leadership of GWO both benefit the subpopulation.

Algorithm 1 presents the pseudocode of the proposed Hybrid Genetic Algorithm–Grey Wolf Optimization (HGAGWO) approach used for PID parameter optimization. The algorithm combines the exploitation capability of GWO with the exploration and diversity-preserving mechanisms of GA. In each iteration, the wolf positions are first updated according to the GWO leadership hierarchy (α, β, and δ wolves). Subsequently, the population is partitioned into several subpopulations, and GA operators, including crossover and mutation, are applied within each subgroup. The newly generated offspring are merged with the GWO-updated solutions, followed by fitness evaluation and elitist selection. The process is repeated until the maximum number of iterations is reached, yielding the optimal PID controller parameters.

**Algorithm 1:**
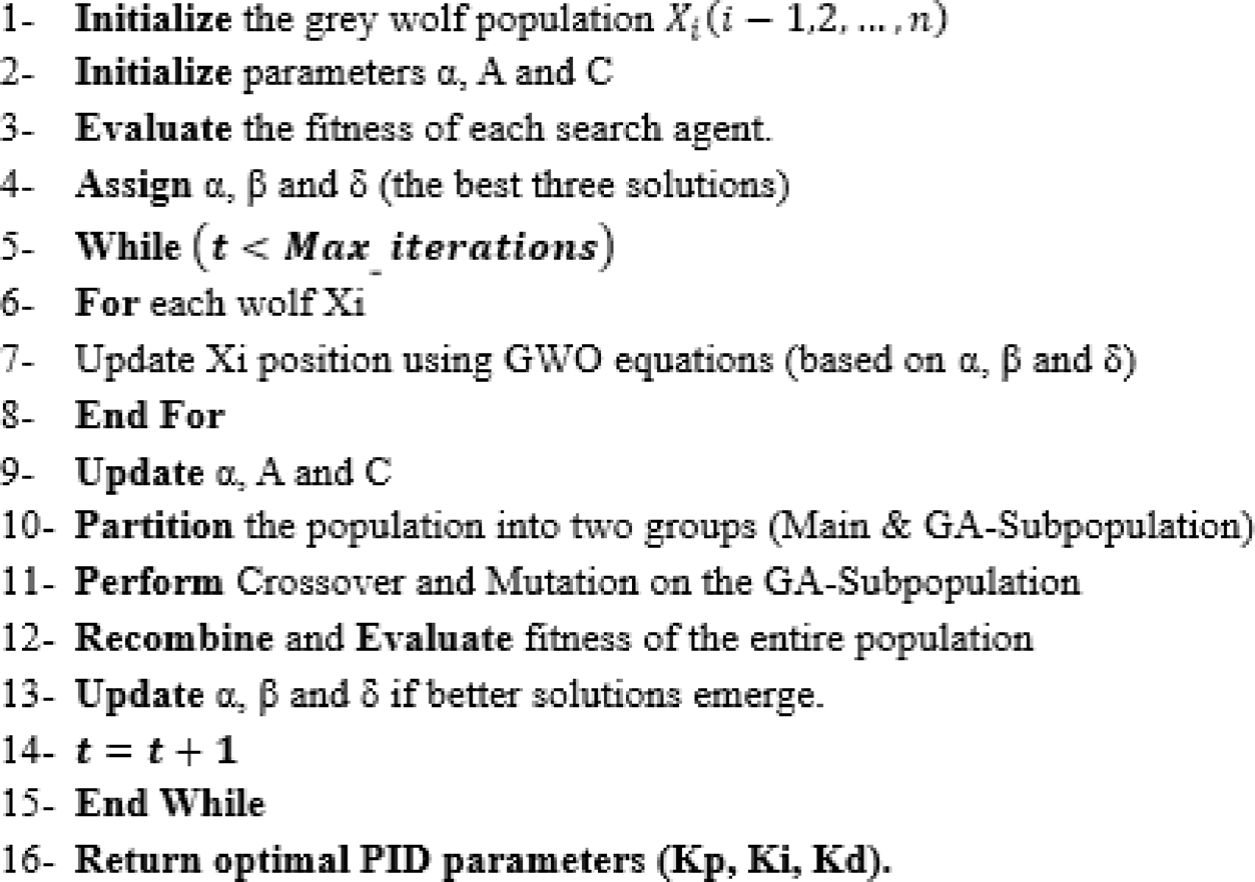
Hybrid GWO-GA (HGAGWO).

## Backlash problem for AC servo motor

There was a problem affecting the accuracy of the servo motor systems^16,34^.

### System model with backlash

**Fig. 9:**
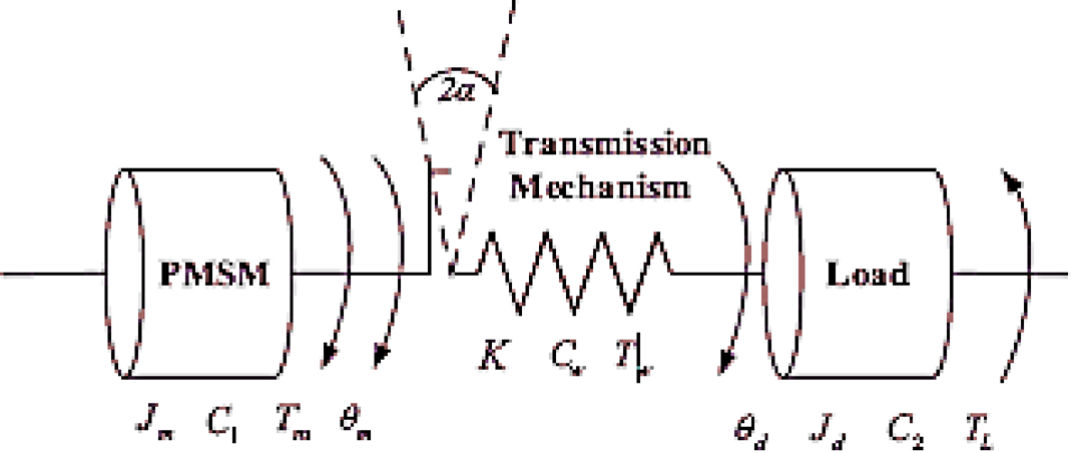
Elastic dual-mass system model with backlash.

One typical nonlinearity in mechanical systems is backlash. Figure 9 shows the elastic dual mass system with backlash model.

13 $$\:\left\{\begin{array}{c}{i}_{d}=\:-\frac{{R}_{a}}{L}\:{i}_{d}+\:{P}_{n}{\omega\:}_{m}\:{i}_{q}+\:\frac{1}{L\:}{u}_{d}\\\:{i}_{q}\:\:=\:-\frac{{R}_{a}}{L}\:{i}_{q}+\:\frac{{P}_{n}\:{\psi\:}_{\:f\:}{\omega\:}_{m}}{L}+\:\frac{1}{L\:}{u}_{q}-\:{P}_{n\:\:}{i}_{d\:\:}{\omega\:}_{m}\\\:{\dot{\omega\:}}_{m}=\:\frac{1.5\:{P}_{n}\:{\psi\:}_{\:f\:\:{i}_{q}}}{{J}_{m}}\:\:-\:\:\frac{{T}_{w}}{{J}_{m}}\:\:-\:\:\frac{{C}_{1}}{{J}_{m}}\:{\omega\:}_{m}\:\\\:{\dot{\theta\:}}_{m}=\:{\omega\:}_{m}\end{array}\right\}$$

Where the currents of d-axes and q-axes are denoted by di and $\:{q}_{i}$ are respectively, $\:{u}_{d}$ as $\:{u}_{q}$ the d-axes and q-axes voltage, respectively, Ra is the resistance, $\:{P}_{n}$ Is the number of pole pairs, L is the inductance, $\:{C}_{1}$ is the viscous friction coefficient of the motor, $\:{\psi\:}_{f}$ is the flux linkage, τ is the external torque on the motor shaft,$\:\:{J}_{m}$ Is the motor inertia, $\:{\theta\:}_{m}$ is the motor angular position, $\:{\theta\:}_{d}$ is the load angle and $\:{w}_{m}$ Is the motor angular velocity^16,34^.

The motor angle and the load angle change by the following angle:

14 $$\:\varDelta\:\theta\:={\theta\:}_{m}-{\theta\:}_{d}$$

$\:{\theta\:}_{w}$ It is assumed to be the backlash angle, and the twist angle of the shaft is

15 $$\:{\theta\:}_{b}=\:\varDelta\:\theta\:-\:{\theta\:}_{W}\left(-\alpha\:\:\le\:{\theta\:}_{b}\le\:\alpha\:\:\right)$$

The shaft torque can be expressed as follows, disregarding the transmission mechanism’s damping coefficient:

16 $$\:{T}_{w}=K{\theta\:}_{W}=\left\{\begin{array}{c}K\:\left(\varDelta\:\theta\:\left(t\right)-\alpha\:\right),\:\varDelta\:\theta\:\left(t\right)\ge\:\alpha\:\:\:\:\:\:\:\:\:\:\:\:\:\:\:\:\\\:0,\:⃓\:\varDelta\:\theta\:\left(t\right)⃓\le\:\:\:\alpha\:\:\:\:\:\:\:\:\:\:\:\:\:\:\:\:\:\:\:\:\:\:\:\:\:\:\:\:\:\:\\\:K\left(\varDelta\:\theta\:\left(t\right)+\alpha\:\right),\:\varDelta\:\theta\:\left(t\right)\le\:-\alpha\:\:\:\:\:\:\:\:\:\:\:\:\:\:\end{array}\right\}$$

**Fig. 10:**
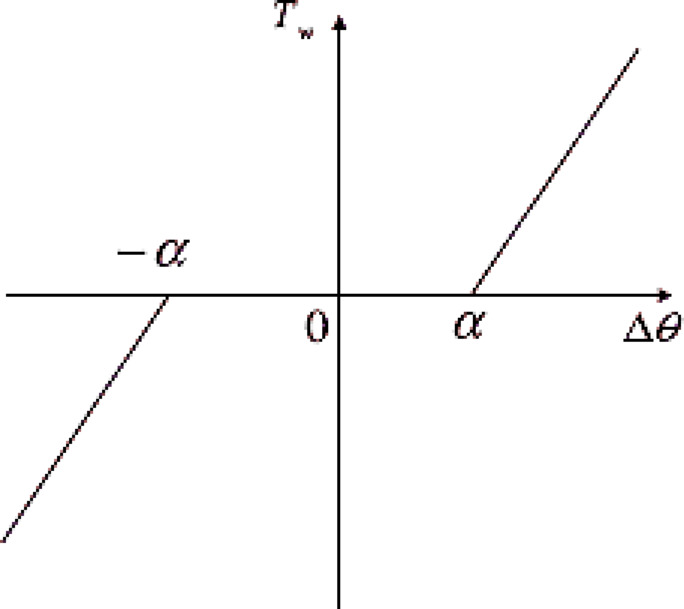
The dead zone model of backlash.

The analysis above indicates that the dead zone model depicted in Fig. 10 can be used to approximate the backlash model. The dead zone model is comparatively simple to examine because it is a static, scalar, non-linear function^16,34^.

Motor side

17 $$\:{\dot{\omega\:}}_{m}=\frac{1.5\:{P}_{n}\:{\psi\:}_{\:f\:\:{i}_{q}}}{{J}_{m}}-\:\:\frac{{T}_{w}}{{J}_{m}}\:\:-\:\frac{{C}_{1}}{{J}_{m}}\:{\omega\:}_{m}$$

Load side

18 $$\:\:\:\:{\dot{\omega\:}}_{d}\:=\:\frac{{T}_{w}\:}{{J}_{d}}-\:\:\frac{{T}_{L}}{{J}_{d}}-\:\frac{{C}_{2}}{{J}_{d}}{\omega\:}_{d}$$

where K and $\:{C}_{w}$are the transmission mechanism’s elasticity and damping coefficient, respectively, and Tm is the motor electromagnetic torque. $\:{\boldsymbol{\theta\:}}_{\boldsymbol{d}}\:$ is the angular position, $\:,\:{\boldsymbol{J}}_{\boldsymbol{d}\:}$inertia, and$\:{\boldsymbol{C}}_{2}$ viscous friction coefficient of the load, $\:{\boldsymbol{T}}_{\boldsymbol{w}}\:$Is the shaft torque, and TL is the load torque (Fig. 10).

### Determining the backlash parameters

Equation (19) states that when $\:\left(\varDelta\:\_\theta\:\:\left(t\right)>\alpha\:\right)$_, the backlash torque $\:{T}_{w}$ in the dead zone is zero. The backlash torque $\:{T}_{w}$ is proportional to the angle difference $\:{\varDelta\:}_{\theta\:}$.

The elastic coefficient K of the backlash model is found using the least squares approach, a conventional offline parameter identification technique. Equation (19) represents the linear regression model at sampling time k.

19 $$\:{T}_{w}\left(k\right)=K\varDelta\:\theta\:\left(k\right)-{K}_{\alpha\:}+{\omega\:}_{s}\left(k\right)\:\:\:\:\:\:\:\:\:\:\:\:\:\:\:\:\:\:\:\:\:\:\:\:\:\:\:\:\:\:\:\:\:\:\:\:\:\:\left(\varDelta\:\theta\:\left(k\right)>\alpha\:\right)$$

where noise was measured by $\:{\boldsymbol{\omega\:}}_{\boldsymbol{s}}$. Equation (20) can be expressed as follows if we select N sets of collected data:

20 $$\:Y=\varnothing\:X+W$$

where the measurement noise is denoted by $\:{\omega\:}_{S}$ If we select N sets of

21 $$\:\left[{\varnothing}=\begin{array}{c}Y=[{{T}_{w}\left(1\right)\:{T}_{w}\left(2\right)\dots\:{T}_{w}\left(K\right)\dots\:{T}_{w}\left(N\right)]}^{T},\\\:{\left[\begin{array}{c}{\Delta\:}\theta\:\left(1\right)\:{\Delta\:}\theta\:\left(2\right)\dots\:\:{\Delta\:}\theta\:\left(k\right)\dots\:\:{\Delta\:}\theta\:\left(N\right)\\\:-1\:\:\:\:\:\:\:-1\:\:\:\dots\:-1\:\:\:\:\:\:\:\:\dots\:-1\end{array}\right]}^{T}\\\:\begin{array}{c}X=\:{\left[K\:\:\:\:\:K\propto\:\right]}^{T},\\\:W={\left[{\omega\:}_{s}\left(1\right)\:\:\:{\omega\:}_{s}\left(2\right)\:\dots\:\:{\omega\:}_{s}\left(k\right)\dots\:{\omega\:}_{s}\left(N\right)\right]}^{T},\end{array}\end{array}\right.$$

We choose the objective function

Min

22 $$\:\:\mathrm{J}\:=\sum\:_{k=1}^{N}({T}_{w}\left(k\right)-\left(\widehat{K}{\Delta\:}\theta\:\left(k\right)\:-\widehat{K}\widehat{\alpha\:}\right){)}^{2},$$

To minimize the objective function, solve the parameter:

23 $$\:\left[\begin{array}{c}\widehat{K}\\\:\widehat{K}\widehat{\alpha\:}\end{array}\right]=({{\varnothing}}^{T}{\varnothing}{)}^{-1}\:\:{{\varnothing}}^{T}\:Y.\:$$

When the motor operates at a constant angle, the backlash torque $\:{T}_{w}$ is equal to the electromagnetic torque $\:{T}_{m}$, and we know that

24 $${T}_{m}=1.5\:{P}_{n}\:{\psi\:}_{\:f\:}{i}_{q}$$

## Simulation results

### Speed control of AC servo motor

Speed control of the AC servo motor by using different controller techniques. The effect of changing the torque on the motor was studied, and then a comparison was made to determine the effect of changing the torque on the servo motor. Speed control of an AC servo motor is a crucial aspect of many industrial applications. Several controller techniques can be used to achieve accurate and precise speed control. The most common techniques include PID control using GA, GWO and HGAGWO. When changing the torque on the motor, the speed of the motor is affected, and this effect can be studied by observing the response of the motor to the change in torque. The motor’s speed can be measured using an encoder or a tachometer, and the torque can be varied by adjusting the load on the motor shaft. Comparing how altering the torque affects the servo motor it is essential to consider the motor’s specifications, including its maximum torque, maximum speed, and inertia. Changing torque will affect the motor’s acceleration and deceleration times, which are crucial in many applications, such as robotics and motion control. To evaluate the controller performance under varying operating conditions, different load torque levels were applied to the AC servo motor, and the corresponding speed and position responses were recorded. The transient response, including rise time, settling time, and overshoot, was then analyzed to assess the effectiveness of the proposed HGAGWO-based PID controller under load disturbances. Beyond this point, the speed will remain constant and further increases in torque will cause the motor to stall or overload. Similarly, decreasing the torque will cause a decrease in the motor’s speed until it reaches a minimum speed, beyond which further reductions in torque will cause the motor to stall or stop. Table 2 shows the AC servo motor parameters.

Table 4 presents the PID gains $\:{K}_{p},{K}_{i}and\:{K}_{d}$ for AC servo motor speed control tuned using different optimization algorithms: GA, GWO, and the hybrid HGAGWO. GA gives relatively high gains for all terms, which means a quick response but possibly more overshoot. GWO gives very low $\:{K}_{p}$and $\:{K}_{d}\:$values with moderate $\:{K}_{i}$, which means a more stable response but slower dynamics. The hybrid HGAGWO achieves a balanced configuration with moderate $\:{K}_{p}$, suitable $\:{K}_{i}$and $\:{K}_{d\:}$set to zero, which means better stability and control accuracy while reducing noise related to derivatives. The hybrid approach effectively balances speed, precision, and robustness, making it perfect for industrial servo applications with nonlinearities like backlash.

**Fig. 11:**
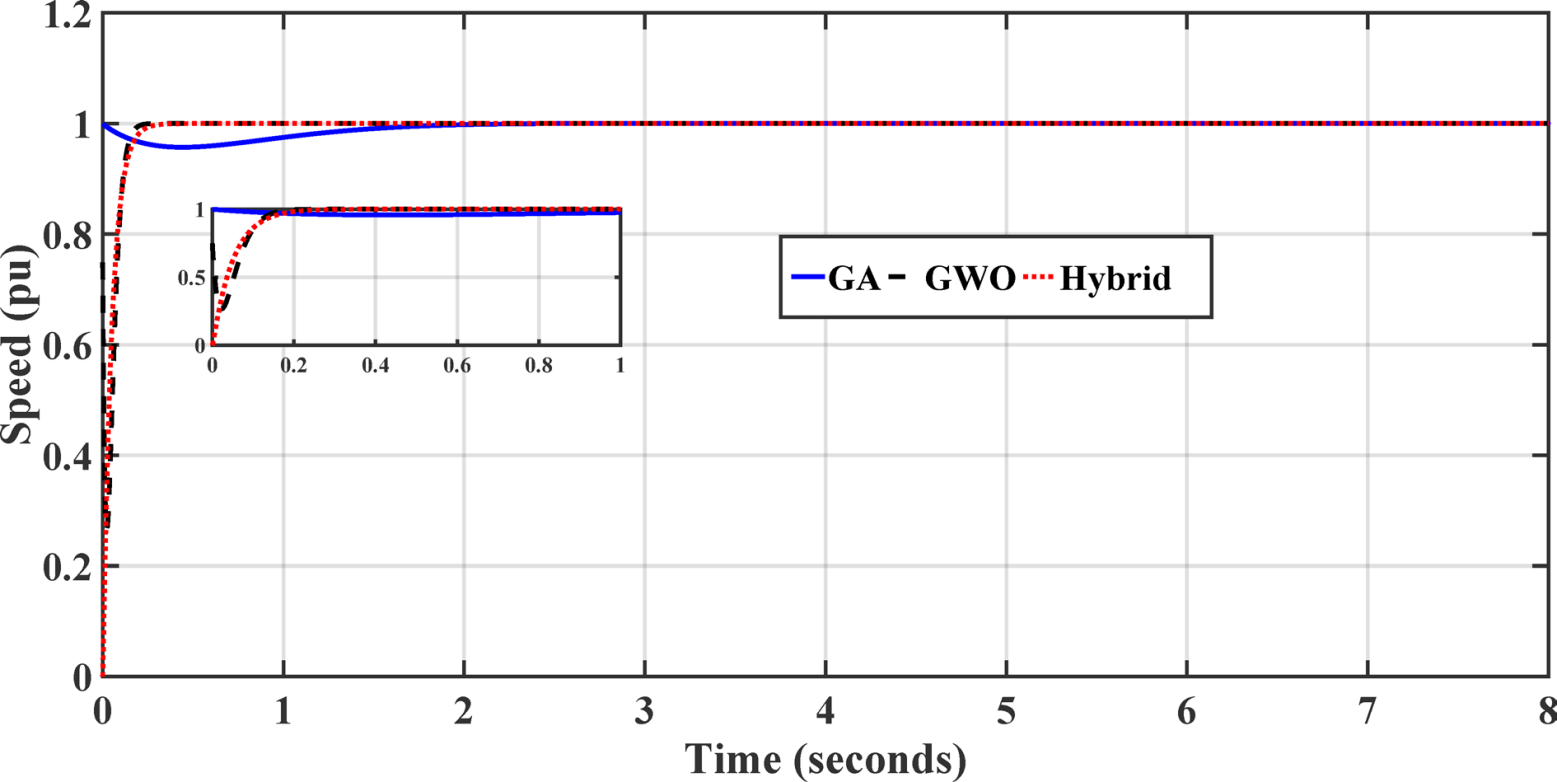
Speed PID controllers of the AC servo motor using GA, GWO, and HGAGWO optimized.

Figure 11 shows the closed-loop speed response of an AC servo motor controlled by PID controllers. The gains of the controllers are adjusted using GA, GWO, and the hybrid HGAGWO strategy. The hybrid approach clearly outperforms the individual GA and GWO methods by achieving the fastest rise time, reduced settling time, and minimal overshoot. The insect emphasizes the initial transient behavior, showing that the hybrid PID controller effectively reduces oscillations while ensuring smooth acceleration toward the reference speed. This improved dynamic response is due to the complementary strengths of GA and GWO; GA provides strong global search capabilities, ensuring convergence toward optimal regions in the solution space, while GWO improves local exploitation by fine-tuning the PID gains to minimize transient errors and overshoot. The results align with contemporary motion control research, which indicates that hybrid metaheuristic algorithms surpass single-strategy optimization in response speed, disturbance rejection, and robustness amid parameter variations. Consequently, the GAGWO tuning strategy offers a highly reliable and precise control solution for industrial applications demanding rapid, accurate, and stable motion control, including robotics and high-performance automation systems.

**Table 4:** PID control benefits for AC servo speed control motors utilizing various optimization algorithms.

Different optimization algorithm	$\:{K}_{p}$	$\:{K}_{i}$	$\:{K}_{d}$
GA	15.3	22.7	4.04
GWO	0.00354	19.6	0.0103
HGAGWO	0.0395	19.6	0

### Position control of AC servo motor

Use several approach controllers to control the AC servo motor’s position. After examining how altering the torque affected the motor, a comparison was made to ascertain how altering the torque affected the servo motor.

Table 5 shows the PID controller gains that were found using three different optimization methods: GA, GWO, and HGAGWO. The values show that each algorithm converges toward a different control strategy. GA produces relatively high proportional, integral, and derivative gains, which leads to a moderately fast response with acceptable stability. GWO converges toward a solution with almost no integral action and very small derivative gain, which shows that the algorithm tends to prioritize fast rise time even if it means a slight steady-state error or overshoot. In contrast, the hybrid HGAGWO method yields a balanced set of gains, keeping a strong proportional component while getting rid of the integral term and applying a moderate derivative gain. This optimized combination improves damping and stability while keeping quick responsiveness.

**Fig. 12:**
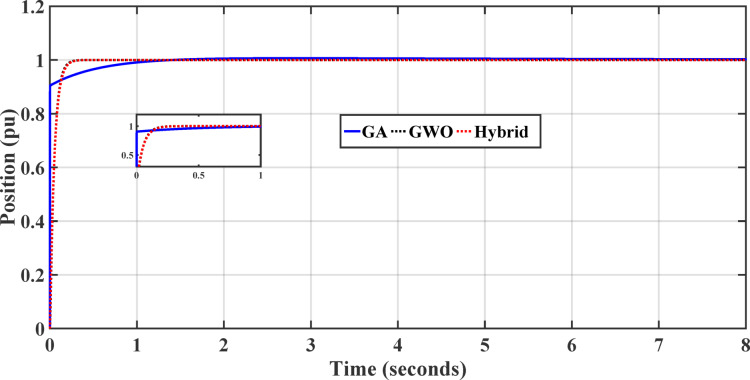
Position PID controllers of the AC servo motor using GA, GWO, and HGAGWO optimized.

Figure 12 shows how the AC servo motor responds to the three optimized PID controllers. The curves show that the hybrid HGAGWO controller has the fastest rise time and the smoothest convergence to the desired position with the least amount of overshoot. The GWO-based controller responds quickly but has noticeable overshoots and a slight steady-state deviation because it doesn’t have much integral action. The GA-based controller has a slower rise time than the other methods, but its settling behavior stays stable. The magnified inset shows even more clearly how well the hybrid controller handles dynamic changes in the early stages of the response. The table and figure both show that the hybrid HGAGWO method is the best way to tune a PID controller, making the servo system’s dynamic performance and positioning accuracy better.

**Table 5:** PID control for AC servo position control motor with different optimization algorithms.

Different optimization algorithm	$\:{K}_{p}$	$\:{K}_{i}$	$\:{K}_{d}$
GA	19.8	3.66	9.45
GWO	19.6	2.74*10^− 6^	0.000769
HGAGWO	19.6	0	0.0564

## Experimental work results

The experimental results demonstrated that implementing PID controllers improved the response characteristics, speed, and accuracy of the AC servo motor. As illustrated in Table 1, the settling time decreased, the overshoot was eliminated, and the rise time decreased, leading to a reduction in the steady-state error. A comparison of various PI and PID controllers on the PLC indicated that the PID controller with the optimal gains yielded the most favorable outcomes. Furthermore, the simulation conducted with MATLAB software corroborated the experimental findings, confirming a significant improvement in the performance accuracy of the AC servo motor.

The proposed control system adopts a cascaded dual-loop architecture consisting of an outer position control loop and an inner speed control loop. The outer loop is implemented in the Siemens S7-1200 PLC, where the optimized PID controller generates the reference speed according to the position tracking error. The generated speed command is then transmitted to the servo drive through the communication interface. The inner loop, implemented within the servo drive, regulates the motor speed using its embedded PID controller to ensure fast dynamic response and disturbance rejection. To improve positioning accuracy, an external encoder is employed to establish a fully closed-loop configuration by providing direct position feedback to the PLC. This arrangement minimizes the influence of transmission backlash and mechanical uncertainties while preventing interference between the two control loops by assigning independent control objectives to each loop.

### Speed control with experimental work

Using various PID and PI controllers, the speed of an AC servo motor can be controlled.

A. **PID controller method**.

**Fig. 13:**
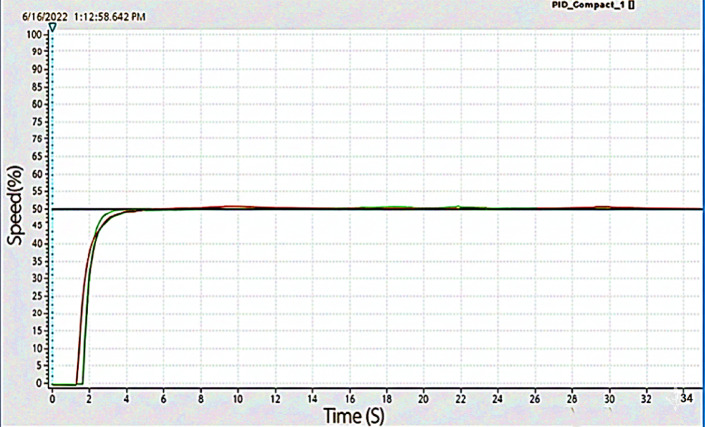
Experimental speed-time curve by using a PID controller.

With the steady state set to 50%, Fig. 13 illustrates the relationship between speed (the current set-point percentage) and time. After 3 s, the value was reached, and after 4 s, the motor curve stabilized. The system was working properly because there were no overshoots, the rising time was rapid, and the motor quickly reached a steady state. However, in general, the performance of a control system, including its stability, overshoot, and settling time, depends on the system’s dynamics, specifications, and requirements. The motor quickly reaching a steady state also indicates that the control system was effective in regulating the motor’s speed to the desired set point value. It is important to note that the performance of a control system can vary depending on the specific requirements and characteristics of the system. For instance, changing the steady-state value can affect the control system’s response, and different controller techniques may be required to meet varying requirements.

B. **PI controller method**.

**Fig. 14:**
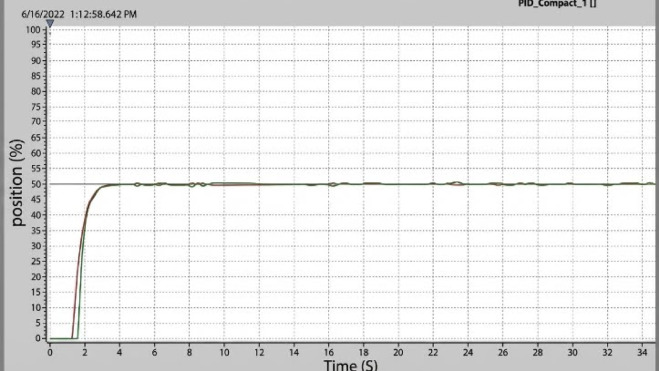
Experimental speed-time curve using the PI controller.

The relationship between speed (current set-point percentage) and time is shown in Fig. 14, with the set point adjusted to 50% of the maximum scale. The rise time was 2.4 s; the system had no overshoot, and the speed reached steady state in under 5 s. The system worked effectively because the motor reached a steady state so quickly and without overshooting, thanks to its fast rise time. Assuming the statements are accurate, the absence of overshoots and the rapid approach to steady state suggest that the control system was well-tuned and provided a stable response. The rapid rise in time may have helped the system avoid overshooting the desired set point. It is important to note that a control system’s performance can vary depending on its specific requirements and characteristics. Changing the set point value can affect the control system’s response, and different controller techniques may be required to meet varying requirements.

### Position control with experimental work

A. **PID controller method**.

**Fig. 15:**
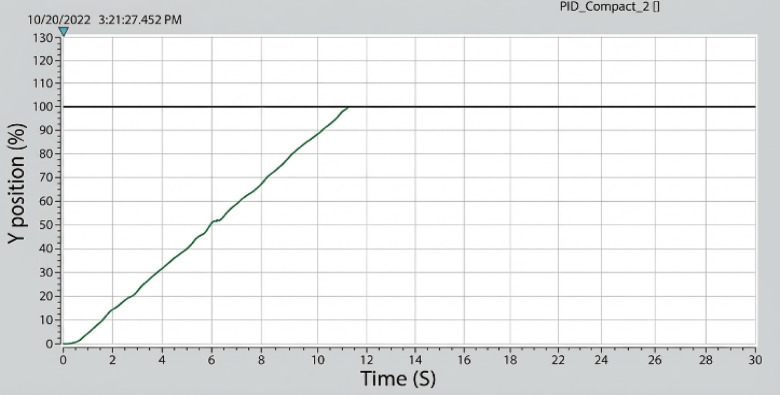
Experimental position with time curve by using a PID controller.

**Fig. 16:**
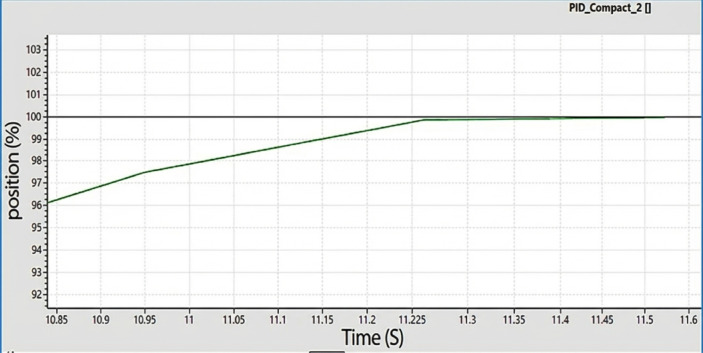
Focus on the steady state for Fig. 16.

In Fig. 15, the motor arrived at a rise time that was very short, and the motor reached steady state quickly and without overshooting. The rising time required 11.25 s to bring the motor to its set point of 100 and reached steady state in just 11.35 s. It is not possible to provide specific feedback on the statements made without seeing Fig. 16. However, in general, the rising time, settling time, and overshoot of a system depend on its dynamics, specifications, and requirements. Assuming the statements are accurate, a rise time of 11.25 s to reach the set point of 100 may be considered slow, depending on the specific system requirements. However, the motor curve’s rapid, overshoot-free approach to steady state suggests that the control system may be well-tuned and stable. It is important to note that the system. A control system’s performance can vary with its specific requirements and characteristics. For different applications and operating conditions, different controller techniques and tuning parameters may be needed. Overall, a thorough analysis and evaluation of the system’s performance and settling times are acceptable and to select the best controller and tuning parameters to ensure stable and effective operation.

B. **PI controller method.**

**Fig. 17:**
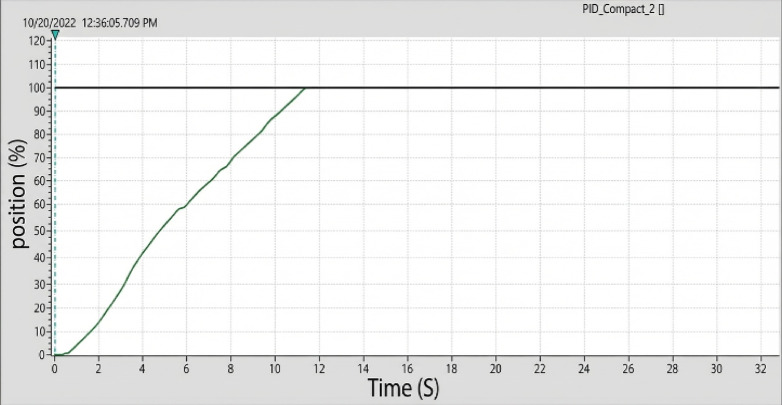
Experimental position-time curve by using the PI controller (current set point%).

**Fig. 18:**
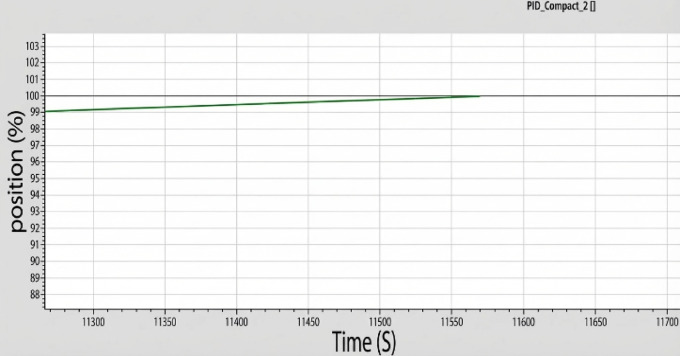
Focus on a steady state for Fig. 18.

As seen in Figs. 17 and 18, the system without overshoot required a 10-second rise time to bring the motor speed to the set point (100), and the motor curve reached steady state after 11.55 s. The system didn’t find an overshoot and quickly reached the goal location in a steady state, increasing system reliability. However, in general, the rising time, settling time, and overshoot of a system depend on its dynamics, specifications, and requirements. Assuming the statements are accurate, a 10-second increase to reach the set point of 100 may be considered slow, depending on the specific system requirements. However, the motor curve’s rapid, overshoot-free convergence to a steady state suggests that the control system may be well-tuned and stable. It is important to note that a control system’s performance can vary depending on its specific requirements and characteristics. Different controller techniques and tuning parameters may be required for various applications and operating conditions.

### Backlash problem position control with experimental work

**Fig. 19:**
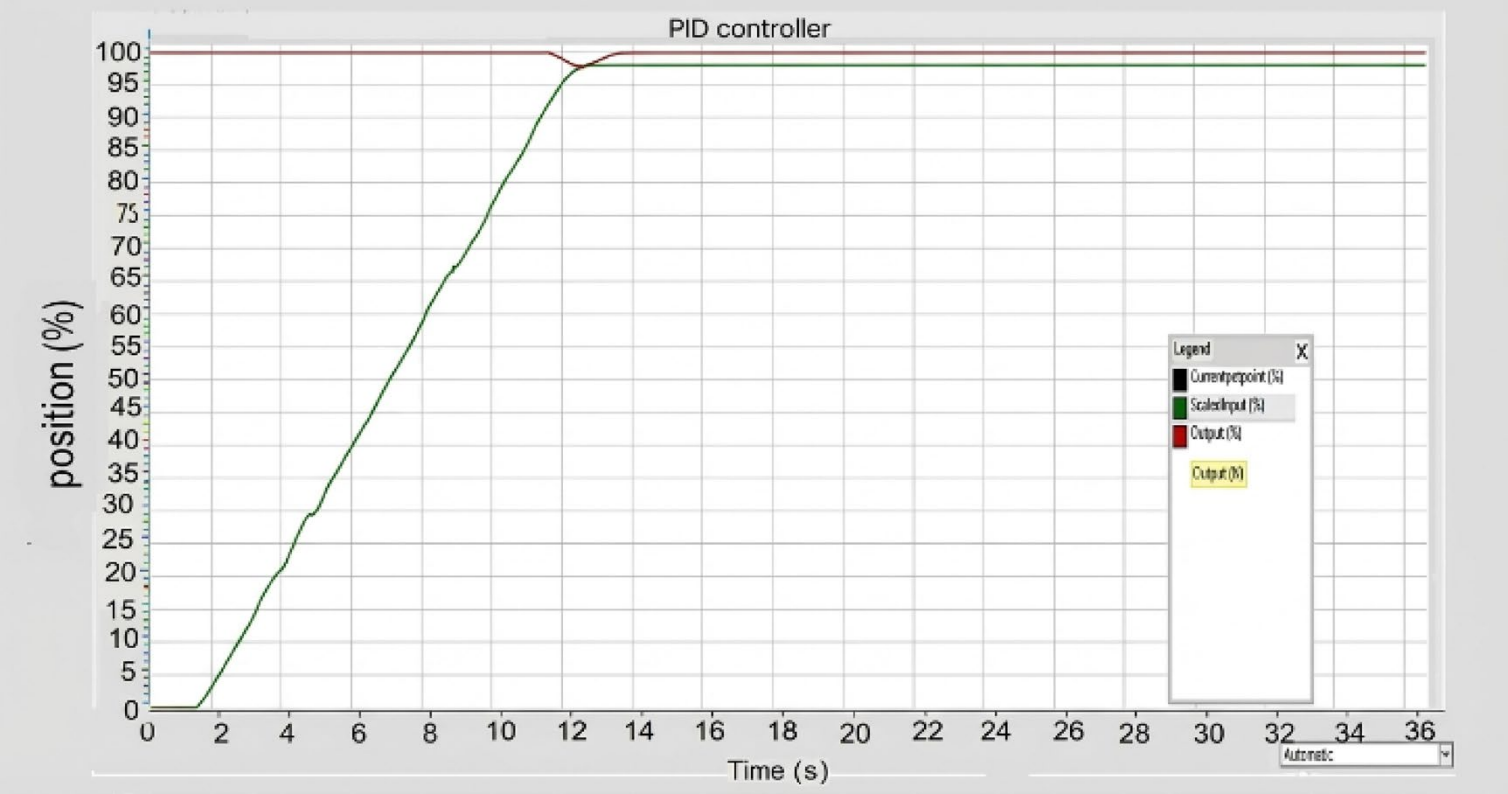
Experimental position by using a PID controller with the backlash problem.

**Fig. 20:**
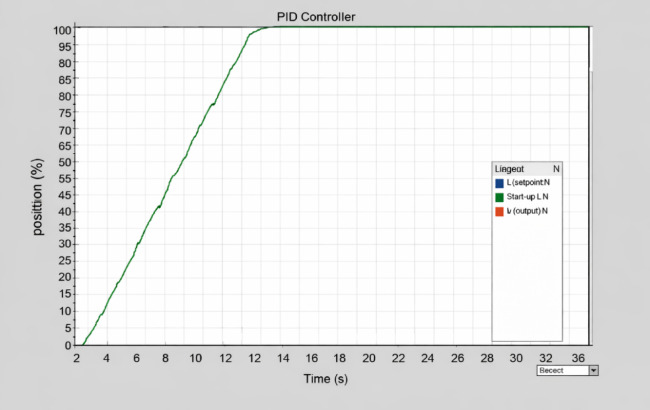
Experimental position using the PID controller after solving the backlash problem.

**Fig. 21:**
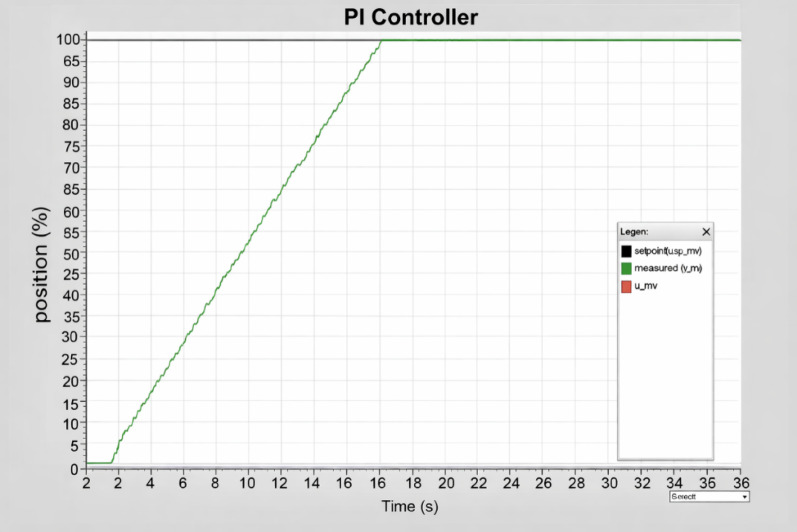
Experimental position using the PI controller with the backlash problem.

**Fig. 22:**
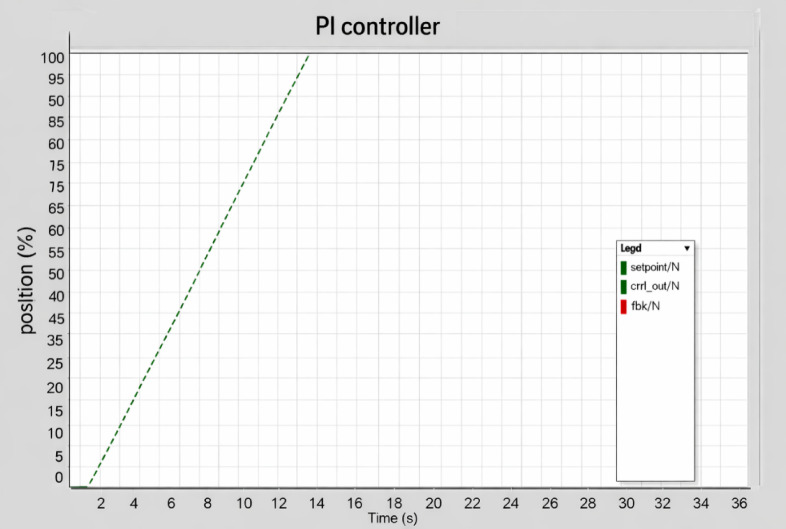
Experimental position using the PI controller after solving the backlash problem.

Figure 19 presents the system’s performance with effective backlash; the rise time was not as fast, and the motor did not reach a steady state. The system is working without overshooting. The rise time required 11.15 s to bring the motor to the speed set point of 100, and the motor’s curve reached a steady state. Assuming the statements are accurate, a rise time of 11.15 s to reach the set point of 100 may be considered slow, depending on the specific system requirements. However, the fact that the motor curve did not reach a steady state in this case suggests that the motor is not working well, and that the system is working without overshooting, suggesting that the control system may be well-tuned and stable. It is important to note that a control system’s performance can vary depending on its specific requirements and characteristics. In Fig. 20, the performance of the system shown is shown after the element of effective backlash. For this problem, the same modification applies is applied to the PID controller parameter and the same change on the compensation value, which parameter from the drive side. After changing the compensation value and PID parameter, the motor reached a steady state. In Fig. 21, the present system with effective backlash reached a steady state after 11 s with a PI controller. The system required 11 s rise time to reach the motor speed at the set point (100). The system worked without overshoot; it didn’t overshoot, and a quick response reached the goal location in steady state, increasing system reliability and stability. Different controller techniques and tuning parameters may be required for different applications and operating conditions. As shown in Fig. 22, the ripple problem was solved for the system before it reached steady state. After changing the compensation value for backlash and adjusting the PI parameter, the motor arrived at steady state.

Furthermore, a significant innovation of our research is its focus on a real-world, industrially relevant challenge the backlash phenomenon in position control systems through a practical experimental investigation using PI/PID controllers optimized with three intelligent tuning techniques: GA, GWO, and the HGAGWO approach. By combining classical control strategies with advanced optimization algorithms and validating their efficacy through experiments on a real servo drive system, the study provides compelling justification for categorizing the proposed methodology within the broader field of intelligent control systems.

Table 6 presents a cross-validation comparison between simulation and experimental results for the PID controllers optimized using GA, GWO, and the proposed HGAGWO algorithms. The results demonstrate a consistent performance trend across both environments, confirming the effectiveness of the proposed optimization approach. In the simulation environment, the HGAGWO-based PID controller achieved the best dynamic performance, with the shortest rise time (0.22 s), lowest settling time (0.45 s), and negligible overshoot (0.2%). The GWO-optimized PID controller ranked second, while the GA-based PID controller exhibited the slowest response and highest overshoot among the three methods. These findings indicate that the hybrid HGAGWO algorithm provides a more effective balance between exploration and exploitation during optimization, resulting in superior PID parameter tuning. The experimental results exhibit longer rise and settling times compared with the simulation results for all controllers. This difference is expected because the simulation model assumes ideal operating conditions, whereas the experimental setup includes practical non-ideal factors such as PLC sampling delays, communication latency, encoder quantization, mechanical backlash, friction effects, actuator nonlinearities, parameter uncertainties, and measurement noise. Despite these implementation constraints, the experimental results preserve the same performance ranking observed in the simulation study. The PID-HGAGWO controller achieved the fastest response, with a rise time of 1.85 s and a settling time of 2.80 s, followed by PID-GWO and PID-GA. Furthermore, all experimental controllers achieved zero overshoot, demonstrating stable operation and validating the effectiveness of the implemented backlash compensation strategy.

**Table 6:** Validation between simulation and experimental performance.

Controller	Environment	Rise Time (s)	Settling Time (s)	Overshoot (%)
PID-GA	Simulation	0.32	0.62	3.8
PID-GA	Experimental	2.00	3.00	0
PID-GWO	Simulation	0.28	0.55	1.5
PID-GWO	Experimental	1.95	2.90	0
PID-HGAGWO	Simulation	0.22	0.45	0.2
PID-HGAGWO	Experimental	1.85	2.80	0

## Conclusions

In summary, this study has introduced a thorough and meticulously validated framework for optimizing the performance of AC servo motor drives via intelligent PID controller tuning, employing GA, GWO, and HGAGWO optimization strategies. The results consistently indicate that the amalgamation of evolutionary and swarm-based optimization techniques results in significant enhancements in dynamic response, steady-state accuracy, and the robustness of both speed and position control loops. Among the evaluated methods, the hybrid HGAGWO approach demonstrates the highest performance, offering expedited convergence, diminished overshoot, and improved stability due to its balanced exploitation traits. A major contribution of this paper is its investigation of the backlash singularity, which is a mechanical nonlinearity that severely limits the accuracy of servo systems. The implemented backlash compensation scheme, which was tested in real time, greatly reduces positioning errors and improves motion fidelity. The strong agreement between simulation results and hardware-based experiments demonstrates that the proposed control architecture is reliable, useful, and feasible for industrial applications. The results show that combining nonlinear compensation with intelligently tuned classical controllers is a strong and scalable solution for high-performance motion control in modern industrial settings This research bolsters the rationale for utilizing hybrid intelligent optimization techniques to tackle real-world uncertainties, nonlinearities, and parameter fluctuations in servo-driven systems. Subsequent investigations may build upon this study by examining adaptive or learning-based enhancements of the HGAGWO framework to further improve system autonomy and real-time performance in intricate and dynamic industrial environments. In experimental work, both controllers reached zero overshoot (0%), which is important for applications that need precise positioning. The PI controller reached the desired position in 10.00 s, while the PID controller took 11.25 s, which is an 11.1% rise time difference. However, both controllers had the same settling time of 11.55 s, which shows that they are both stable over the long term.

Although the proposed HGAGWO algorithm demonstrated superior performance in both simulation and experimental investigations, future work will include extensive statistical validation through multiple independent optimizations runs, confidence interval analysis, and non-parametric significance testing to further quantify the robustness and repeatability of the obtained solutions.

## Data Availability

Concerning the availability of raw data, I want to inform you that this work is part of a long research study, and the raw data will not be public now, but are available from the corresponding author upon request.
